# A systematic literature review for APT detection and Effective Cyber Situational Awareness (ECSA) conceptual model

**DOI:** 10.1016/j.heliyon.2023.e17156

**Published:** 2023-06-16

**Authors:** Duraid Thamer Salim, Manmeet Mahinderjit Singh, Pantea Keikhosrokiani

**Affiliations:** a School of Computer Sciences, Universiti Sains Malaysia, Penang, 11800, Malaysia; b Department of Computer Science, College of Basic Education, Mustansiriyah University, Baghdad, Iraq

**Keywords:** APT malware, Network traffic, Intrusion detection system, Machine learning, Attack behaviour, Situation awareness model

## Abstract

Advancements in computing technology and the growing number of devices (e.g., computers, mobile) connected to networks have contributed to an increase in the amount of data transmitted between devices. These data are exposed to various types of cyberattacks, one of which is advanced persistent threats (APTs). APTs are stealthy and focus on sophisticated, specific targets. One reason for the detection failure of APTs is the nature of the attack pattern, which changes rapidly based on advancements in hacking. The need for future researchers to understand the gap in the literature regarding APT detection and to explore improved detection techniques has become crucial. Thus, this systematic literature review (SLR) examines the different approaches used to detect APT attacks directed at the network system in terms of approach and assessment metrics. The SLR includes papers on computer, mobile, and internet of things (IoT) technologies. We performed an SLR by searching six leading scientific databases to identify 75 studies that were published from 2012 to 2022. The findings from the SLR are discussed in terms of the literature's research gaps, and the study provides essential recommendations for designing a model for early APT detection. We propose a conceptual model known as the Effective Cyber Situational Awareness Model to Detect and Predict Mobile APTs (ECSA-tDP-MAPT), designed to effectively detect and predict APT attacks on mobile network traffic.

## 1 Introduction

Data are individual facts, statistics, or items of information, often numeric, and are considered the new gold [1]. With the advancement of computing technology, data are being produced, processed, and used in several computer and mobile services. In 2021 [2], the number of internet users increased by 7.6%, and currently 60% of the world's population is online. The volume of data created every day is approximately 1.145 trillion megabytes. In networking, data are divided into packets. A packet is a small part of a larger message that is directed to devices such as smartphones and computers and that contains control and user data [3]. User data carry content (e.g., a document, PDF, URL, or attachment), which is known as the payload. In contrast, control data provide information about how to get the payload to its destination (e.g., network addresses for the sender and receiver, codes for error detection, and the sequence of packets or data). Control data are usually found in the headers and trailers of the packet [3]. Ethernet packets are around 1.5 KB and IP payloads are 64 KB. Network traffic refers to the packets that traverse a network. However, not all packets are useful or safe. To compromise or overwhelm a network, attackers can generate malicious network traffic.

Due to the growing need for a mobile and wireless network environment, smartphones have become essential. The data stored in a smartphone include personal and financial information, the user's location, and call logs [4]. Besides being mobile, smartphones are also small, with limited resources [5] and heterogeneous services [4] running on them. Thus, user data stored in smartphones is prone to being leaked and compromised due to the lack of security mechanisms. As a result, smartphones have become a popular target for cyberthreats [5], such as advanced persistent threats (APTs).

An APT is a stealth threat actor that obtains unauthorised access to a device network [6]. Attackers may use well-known strategies (e.g., spear phishing, SQL injection, malware, watering holes, and repackaging) to hack the target entity's network, but the tools (e.g., AndroRAT [7] and Dvmap [8]) used to penetrate the systems are advanced and can avoid detection. They stay hidden, moving from system to system inside the organisation's network, gathering valuable data. APTs are typically carried out by well-sponsored attackers who are given the resources necessary to carry out the attack for an extended period. According to the US National Institute of Standards and Technology (NIST) [9], the number of malicious installation packages discovered on mobile devices increased by approximately 1.5 million from Q4 2015 to Q1 2021. APT attacks cause financial loss, intellectual property theft, information leakage, and other problems. According to research by the National Association of Resilient Insurers (NAR) and the US Department of Homeland Security (DHS) [10], the APT prevention market for cyberattacks is anticipated to be valued at more than 12.5 billion US dollars annually by 2025.

APTs use spear-phishing and watering-hole attacks, which are forms of social engineering, to obtain data from the target network [11]. Spear phishing targets specific people or groups within a company. It starts with the attackers sending malware-infected emails. These fake emails are crafted to entice targeted receivers to open attachments. Watering-hole attacks, unlike spear-phishing attacks, involve infecting a website that employees of the target organisation frequently visit. Once the attacker sends payloads to a compromised device to establish a channel of command and control (C&C), the APT attackers can create long-term connections to steal sensitive data. An attacker will compress and encrypt stolen sensitive data before exfiltrating it to avoid detection or make the exfiltration less obvious to a defender [12].

Outmoded cybersecurity protocols such as firewalls and intrusion detection systems (IDSs) cannot thwart APT attacks because those attacks use social engineering to trick unsuspecting humans into giving access to attackers. Because APT attacks are constantly changing and subtle, traditional cyberthreat defences are rendered useless when facing APT attacks [11]. There are considerable challenges to detecting APT malware in a network environment:

(i) With increasing network traffic and an increasing number of connected devices, it is difficult to discover APT attacks in a timely way [6]. Since the volume of network traffic is huge and entails the use of many resources, it becomes difficult to monitor events in the whole network system [6]. A networking event that involves the transmission of traffic sent and received may be APT attacks and have malware within them. APT malware patterns are dynamically changing due to advancements in hacking tools and techniques. When the traffic grows exponentially, the detection engine needs to function faster and be capable of effectively detecting the attack. This is possible only when the detection engine becomes more responsive to attack-pattern transformation.
(ii) In the case of a secure internet connection using HTTPS, most packets are secured with encryption [13], a key technology in the various privacy-enhancing tools that have appeared in recent years. Tor is a browser with a sound privacy protection mechanism [13]. By encrypting and tunnelling traffic through a distributed network of servers, Tor allows the attacker to hide their identity and internet activity. For this reason, some network traffic on that protected internet communication channel is also encrypted.
(iii) IDSs analyse attack behaviour but are inefficient at detecting multistep attacks. Due to the complex nature of APT, attacks can be uncovered only by uncovering their many life cycle stages. However, most studies have focused on only one stage of an APT. Therefore, detecting an APT technique is unlike detecting an APT attack. In addition, anomalies are not always an indicator of an APT attack, and benign anomalies may increase the false-positive rate [14].

Based on the current challenges in detecting APT malware in a network environment and after identifying closely related studies, we conducted this systematic literature review (SLR) to gain a clear view of the research on APT detection mechanisms that have been published in recent years. This SLR is structured as follows: after the introduction, the study's background is presented in section two of this paper. Section three presents the research methodology, which includes research questions and inclusive review protocols. Section four displays the analysis and findings. Section five discusses the study directions and is followed by concluding remarks in the final section.

### 1.1 Objectives and contributions of study

This study analysed APT detection mechanism research and found the optimal algorithms, architectures, frameworks, and models for different scenarios. This study's objectives were to.

(1) provide an SLR on APT detection and prediction mechanisms,
(2) provide an analysis that characterizes and assesses the machine learning (ML) techniques used for APT detection on network systems, and
(3) Identify research gaps and provide essential recommendations based on the literature while suggesting areas for future study.

This study contributes to providing background on and a comprehensive overview of the most up-to-date literature on APT detection mechanisms in network traffic, which can help security experts and researchers understand APT techniques. In addition, it summarizes the current state of this field of study and offers recommendations for future research. As a recommendation for improving the APT detection process, a conceptual model is also proposed to detect APT attacks for mobile platforms effectively.

### 1.2 Related work

As seen in Table 1, SLRs have significantly contributed to the literature on APTs. Hussain et al. [15] provided a broad overview of APTs and their communication mechanisms, which involve compromised hosts communicating with C&C servers that issue commands and exfiltrate data using persistent malware. The authors also analysed several APT detection frameworks and covered eight papers published between 2011 and 2017, highlighting their limitations. Additionally, they proposed a multilayer protection and detection system for industrial control systems that targets one stage of an APT lifecycle, specifically a C&C stage, to enhance network security.

**Table 1:** A summary of the Variations between the frameworks or models proposed in previous systematic literature reviews and the model proposed in this paper.

References	No. of studies	Duration	Framework/model
Hussain et al. [15]	8	2011–2017	Industrial control system APT defence framework
Jabar and Singh [16]	112	2011–2022	A conceptual framework for identifying and mitigating the severity of abnormal activities across the entire APT lifecycle
Talib et al. [17]	122	2007–2022	Not proposed
Kotenko et al. [18]	127	2010–2021	Not proposed
Khalid et al. [19]	48	2017–2022	Not proposed
Model proposed in this paper	75	2012–2022	Effective Cyber Situational Awareness Model to Detect and Predict Mobile APTs (ECSA-tDP-MAPT) based on network traffic

Jabar and Singh [16] investigated and assessed various defence mechanisms against APTs on mobile devices and networks. Moreover, the authors suggested a conceptual framework to identify suspicious events in all APT life-cycle phases and mitigate their severity through continuous mobile device behaviour monitoring. Their study covered 112 papers published from 2011 to 2022.

Talib et al. [17] presented a comprehensive analysis of potential APT beaconing detection solutions that can ensure the safety of target organisations. They focused mainly on techniques and strategies that detect C&C malware and beaconing during a targeted APT. In addition, their study covered 122 research papers, and they covered 31 APT and beaconing detection vendor projects between 2007 and 2022.

Kotenko et al. [18] provided a broad overview through 127 articles published between 2010 and 2021 in the field of security-event correlation and the approaches used to correlate individual events and their sequences in different attack scenarios, such as APT attacks, with the possibility of detecting an unknown attack, architectural solutions, and the use of initial event data. They further described the data set and the metrics used to assess event-related approaches. They also identified existing issues and potential methods to overcome them.

Khalid et al. [19] analysed 48 articles published between 2017 and 2022 about game theory approaches to addressing APTs. They found that game theory provides a framework for understanding and analysing strategic interactions between defenders and attackers, optimizing defensive performance, and implementing security measures to anticipate and prepare for countermeasures. They identified APTs' challenges, such as how tactics and techniques can evolve to bypass defences to avoid detection.

We have investigated and evaluated 75 papers published between 2012 and 2022 in-depth to identify and evaluate detection mechanisms used against APTs in a network environment using ML techniques. The SLR also provides an overview of the tools and methods used to monitor incoming and outgoing network traffic to detect APT attacks. Finally, we propose the Effective Cyber Situational Awareness Model to Detect and Predict Mobile APTs (ECSA-tDP-MAPT) conceptual model, designed to detect and predict mobile APTs based on network traffic effectively.

## 2 Background

This section addresses the general characteristics associated with detecting APT attacks due to vulnerabilities in network devices.

### 2.1 APTs

US Air Force analysts coined advanced persistent threats in 2006 to describe intrusive practices [20] targeting civilians. Thus, military teams could analyse the attack without revealing the identities of those involved. The APT attacker is a highly skilled and resourceful adversary capable of exploiting multiple attack vectors to achieve their goals.

An APT attack is sophisticated and tailored to the target's vulnerabilities. Currently, several of those threats remain undetected. Once detected, they reappear and are modified so the attacker can achieve their goal. FIN6 [21], APT10 [22], and APT41 [23] are examples of groups that have conducted attacks that caused important losses of funds, confidential data, and intellectual property.

Table 2 demonstrates the core variances between traditional attacks and APT attacks in four areas: actors, victims, objectives, and approach. APT actors are typically skilled hackers with ample financial and technical resources that work in teams, but in a traditional attack, a single actor executes the attack. Victims are specific in APT attacks, which often target organisations, governmental institutions, and commercial enterprises. In contrast, in traditional attacks, victims are unspecific, and the attacks target individual systems. APT attacks have clear objectives. Most of the time, the targets are governments or organisations with valuable intellectual property, trade secrets, and so forth, whilst traditional attacks are typically carried out to get personal information, such as numbers of credit cards, to extract money. Finally, APT attacks are designed to be undetected, blend into network traffic, and interact only sufficiently to reach their objectives. In contrast, traditional attacks usually employ ‘smash and grab’ strategies that alert defenders.

**Table 2:** Variations between traditional and APT attacks.

Traditional attacks	APT attacks
Mostly target a single actor	Conducted by a group of skilled hackers that is well-resourced from both monetary and technical viewpoints
The victim is unspecific, and attacks target individual systems	The victim is specific, and attacks target organisations and governmental institutions
Aim for financial gain	Aim to achieve the theft of intellectual property and provide strategic benefits
Usually employ ‘smash and grab’ strategies that alert defenders	Designed to be undetected and blend into network traffic

#### 2.1.1 APT attack stages

**Fig. 1:**
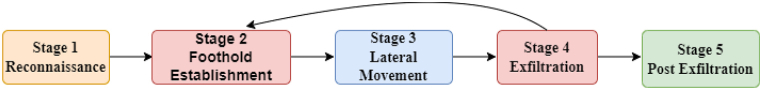
Stages of APT attack.

The attackers move through many stages of attack while remaining hidden. Fig. 1 displays the typical stages of an APT attack. The attack has five stages, including reconnaissance to gather information, the establishment of footholds that employ social engineering methods to penetrate the targeted system, and scanning internal networks for vulnerabilities that might not be apparent from outside the network but allow for reaching the target system. If an attacker gains access to sensitive data on the network, they will use covert communication channels to exfiltrate that data. Finally, after achieving their goal, the attacker may leave backdoors for future attacks.

APT attackers differ based on the intent or motive of their attack. Some attacks are for financial gain, espionage, sabotage, or various interests attackers may have. Therefore, APT attackers can be categorized as attackers who (i) are government-sponsored and target other countries to gain access to sensitive information, such as military or government secrets; (ii) seek financial or personal gain by having access to sensitive information and using their privileged access to steal or leak that information; (iii) aim to achieve political or social goals by targeting organisations or governments that they perceive as oppressive or unjust; and (iv) are employed by rival organisations and target their competitors to gain a competitive advantage by stealing valuable information such as trade secrets, research and development data, or customer data.

#### 2.1.2 Impact of APTs

APTs are sophisticated threats that try to steal data or harm a target network system over time and that target government and commercial enterprise networks [24]. An APT is a threat actor that obtains unauthorised access to a computer network stealthily. By stealing, eavesdropping, or disrupting, the threat actor intends to cause harm to the organization and obtain sensitive data. Because an APT attack typically remains undetected for an extended period, the attackers have ample time to complete the attack cycle and accomplish their goal. The APT protection market is forecast to exceed 15 billion US dollars by 2026 [25]. Originally, APTs primarily targeted personal computers (PCs). However, experts have recently discovered APTs that target mobile devices. Table 3 shows numerous cases of APT attacks on computers or mobile devices.

**Table 3:** Real cases of advanced persistent threat (APT) attacks.

APT case	Platform	Target	APT technique	Impact
Stuxnet [26]	Computer	Iran's uranium nuclear project	Malware	Disturb critical components
CloudAtlas [27]	Mobile	A civil servant, an oil and financial CEO	Application repackaging	Leak sensitive information
Stealth Mango and Tangelo [28]	Mobile	Military, medical, and civilian personnel in Pakistan, Afghanistan, India, Iraq, Iran, and the UAE	Watering hole	Leak sensitive information
Carbanak [29]	Mobile	Banking/financial institutions	Spear phishing	Steal sensitive information
Hydraq [30]	Computer	Google	Malware	Steal organizational data
Marcher [31]	Mobile	Customers of Bank Austria, Raiffeisen Meine Bank, and Sparkasse	Spear phishing	Steal sensitive information
TwoSail Junk [32]	Mobile & computer	Users in Hong Kong	Watering hole	Cyber espionage
Transparent Tribe [33]	Mobile & computer	Government entities, military (Afghanistan, India, and Pakistan)	Spear phishing	Cyber espionage, data theft
Mata [34]	Computer	Corporate entities (Germany, India, Japan, Poland, South Korea, and Turkey)	Malware	Steal customer databases and distribute ransomware
DeathStalker [35]	Computer	Financial technology companies, law offices, wealth consultancy firms, financial sector	Spear-phishing emails	Steal sensitive business information
ZooPark [36]	Mobile	Focus on the victims of Middle Eastern targets in Egypt, Jordan, Morocco, Lebanon, and Iran.	Watering hole	Steal sensitive information
FinSpy [37]	Mobile & computer	Activists, criminal suspects (Vietnam, Canada, Germany, Indonesia, Japan, Mongolia, Russia, and Ukraine)	Malware	Surveillance
Adwind [38]	Mobile & computer	Financial institutions; government entities; healthcare, manufacturing, mass media and TV, shipping, software companies; telecoms, commerce (Germany, Hong Kong, India, Italy, Russia, Taiwan, Turkey, USA)	Spear phishing	Cyber espionage, surveillance
Hacking Team RCS [39]	Mobile & computer	Activists, criminal suspects, journalists, politicians (Germany, India, Iraq, Italy, Mexico, Russia, Turkey, Ukraine, and Vietnam)	Malware	Surveillance
Desert Falcons [40]	Mobile & computer	Victims representing the military and government, to targets of leading media entities and financial institutions	Spear-phishing emails, watering hole	Cyber espionage, data theft, surveillance

Attackers gather data on the targeted entity to identify and exploit vulnerabilities to reach their goal. They then move inside the network, activating privilege escalations as needed until they gain access to sensitive data, which they then send to the attackers' C&C centre via the internet. At the same time, APT attackers employ many techniques, such as spear phishing, SQL injection, malware, watering-hole attacks, and application repackaging, to access victims’ devices. That shows that attackers exploit user trust in these cases [41].

Furthermore, the APT types or vectors most attackers use are as follows: (i) Spear phishing targets specific people or groups within a company. It occurs in the initial stage of an APT attack. It starts with the attackers sending malware-infected emails [35]. These fake emails are crafted to entice targeted receivers to open attachments to trick them into disclosing sensitive information or installing malware. (ii) Watering-hole attacks, unlike spear-phishing attacks, involve infecting a website that employees of the target organization frequently visit. A watering-hole attack occurs in the early stages of an APT attack. Once the attacker sends payloads to a compromised device to establish a channel of C&C, the APT attacker can create long-term connections to steal sensitive data [36]. (iii) Malware is software designed to steal sensitive information, install additional malware, or compromise the target's network [37]. It occurs in the early stages of an APT attack. Malware can be sent via spear phishing, USB devices, and web downloads. (iv) Application repackaging occurs in an APT attack's early or middle stage. An attacker creates a malicious version of a legitimate app by obtaining it from a distribution platform (e.g., the Google Play Store or the Apple App Store). The attacker then adds malicious features and returns the modified app to users who think they are using the original app [41]. Malware is introduced to the victim's device when they download and install the app.

### 2.2 Threat modelling

Threat modelling (TM) is part of risk modelling. In this ongoing process, an asset is first defined and profiled; then identified, prioritized, and monitored as a cyberthreat; and, finally, assessed according to its associated controls [42]. The objective is to identify, classify, and describe threats that reveal an assailant or a campaign of attacks. Resilience is built by anticipating, withstanding, and recovering from security incidents [43]. TM has 10 approaches, including the Cyber Kill Chain model [44]; MITRE's Adversarial Tactics, Techniques, and Common Knowledge (ATT&CK) framework [12]; the National Institute of Standards and Technology (NIST) guidelines [45]; and attack trees [46].

#### 2.2.1 Cyber kill chain model

The kill chain is a military concept that refers to the sequence of events during an attack [42]. The goal is to defend against or exploit the opponent's attacks as they go through their different stages of life [44]. Reconnaissance, weaponization, delivery, exploitation, installation, C&C, and actions on objectives (AOO) are the stages of the intrusion kill chain [44]. Identifying cyberthreats via attack patterns and the various stages in their ‘kill chain’ is necessary for an effective response. The tactics, techniques, and procedures (TTP) employed by an APT are attack patterns. Tactics are the objectives or states an attacker seeks to achieve to accomplish their mission. A technique is a method by which a behaviour achieves a goal or state [47]. The procedure is a list of APT tasks at each life-cycle stage. An APT can use a variety of tactics to complete each of these tasks. Each of these tactics employs one or more techniques.

The Kill Chain model deconstructs and identifies the pertinent characteristics of any complex attack. For example, kill chain analysis has been used to analyse APT attacks such as Stuxnet [26] and Desert Falcons [40] by breaking down the attack process into different stages or steps. The model allows analysts to identify the TTPs used by the attackers at each stage and understand the sequence of events that led to the successful compromise of the target system. For example, in the case of Stuxnet, the Kill Chain analysis revealed that the attackers used multiple zero-day exploits and social engineering tactics to deliver the malware to the target system, followed by a series of steps to propagate the malware and execute its payload. Similarly, in the case of Desert Falcons, the Kill Chain analysis showed that the attackers used spear-phishing emails and social engineering tactics to lure the victims into downloading and executing malware, which was then used to steal sensitive information and spy on the victims.

#### 2.2.2 MITRE's ATT&CK framework

MITRE's ATT&CK is a framework, curated knowledge base, and model for cyber adversaries' actions. It shows the attack phases and platforms known to be targeted [12]. MITRE had enterprise assets (Windows/macOS/Linux), mobile devices, and PRE-ATT&CK matrices before October 2020 [42]. PRE-ATT&CK covers reconnaissance, weaponization, and delivery and is included in Version 8 of the ATT&CK Enterprise Framework. It works more efficiently with all stages of a kill chain, from post-access utilization and installation to C&C and AOO [42]. An adversary's tactics are their specific goals for the operation. An ATT&CK model's techniques describe how an opponent might achieve their tactical objectives. ATT&CK builds on a Cyber Kill Chain by focusing on these adversaries' tactics, techniques, and indicators of compromise (IOC). Several ATT&CK techniques, unlike IOCs, are jobs of the legal system that can be abused and makes it more difficult for defenders to discover them [42].

MITRE ATT&CK methodologies and procedures give behavioural observables for detecting attacks by studying cyber artefacts gathered from the network and end systems. Because of TTP's organisation, analysts can categorize antagonistic acts according to the procedures associated with various techniques and tactics. Analysts can use this information to better prepare for potential attacks and countermeasures. MITRE ATT&CK describes various possible attacks, but it lacks any guidance on how an adversary may combine these attacks to succeed. Therefore, analysts need to employ targeted methods when building TTP chains. Technique associations significantly improve the analyst's capacity to think critically about malicious behaviour and forecast upcoming techniques using the observed ones in the TTP chain [47].

The ATT&CK framework addresses strategic and operational intelligence deficiencies with a quantitative data model. Strategically, the executive leadership utilises actionable intelligence to prioritise and maximise resources while minimizing risk. This framework aids in threat analysis, vulnerability management, and security awareness at the operational level [42] based on the current threat landscape.

#### 2.2.3 NIST guidelines

The NIST has a draught guide (800–154) for data-centric threat modelling systems. It discusses a four-step qualitative methodology for threat modelling [45]. The identification and characterization process is the first step. It contains only data unique to a single host or a small set of related hosts and devices. Based on risk assessments, the second step is determining where an attacker might try to get in (probability and impact). Third, particular attack behaviour and patterns are mitigated by security controls. Controls for risk mitigation are determined. In the final step, the threat model is considered to identify attack vectors and controls for risks that cannot be accepted [42].

#### 2.2.4 Attack trees

Diagrams that are data hierarchical in nature are known as attack trees [42]. It means they draw threats and vectors of attack to realize their objective. A cyberthreat model is a concept introduced by Bruce Schneier. It assigns risk and cost to all known system attacks [46]. Each attack vector is categorized and assigned risk and cost values by attack trees [46]. Defining the primary objective and decomposing it into sub-objectives are typical attack tree stages. The root node represents the objective of the attack, while the leaf nodes represent the various routes that can be taken to achieve that objective [42].

#### 2.2.5 Data flow diagrams (DFDs)

DFDs are graphical representations of the system's inputs, logical processes, and outputs. They focus on trust boundaries, external entities, data storage, processing, and data flow. Making a DFD takes time, and it should not be used alone. A DFD is only one stage of a threat modelling process [42].

#### 2.2.6 Spoofing, tampering, repudiation, denial of service, and elevation of privilege (STRIDE) model

The STRIDE model is a taxonomy that relies on a system or software to identify threats by type. Introduced in 1999, it helps Microsoft developers identify software threats. A design flaw, a coding bug, or an insecure configuration can be the root cause of a data breach [48]. STRIDE mitigates risks associated with confidentiality, availability, authorization, authentication, and nonrepudiation [42]. Threats may have multiple STRIDE categories, or a threat may have several STRIDE categories.

#### 2.2.7 Stochastic or mathematical models

The most common way to do stochastic (or mathematical) threat modelling is to turn attack actions and attributes into Markov chains and then use state transition matrices to analyse them. Thus, the system's next state is determined by its current situation. An attack's current path requires past and present occurrences. This feature allows Markov chains to find attack vector chains that use both events [49]. Cyberthreats such as APT have been modelled using the concept of game theory. Game theory is used to construct a multiphase Bayesian game framework to gather incomplete data regarding deceptive APTs and their multiphase movements [42].

#### 2.2.8 Common attack pattern enumeration and classification (CAPEC) database

CAPEC is an unusual vulnerability database. It is a set of the most common ways that attackers have used common weakness enumerations (CWEs). It analyses and categorizes cyberattacks into before- or after-exploitation attack patterns. It also documents popular cyberattacks and the procedures for their mitigation. The CAPEC model has three levels (standard, meta, and detailed) [50]. The attack patterns hackers use to exploit vulnerable systems are characterized by certain behaviours and techniques. The first kind of pattern is a meta-attack pattern, which does not give specific details about how cyberattacks are made or how they work. The second category consists of prevalent attack patterns, which are more systematic and specific. The third pattern is the attack pattern in detail. This specific pattern provides extensive detail, including any associated or supporting detailed attack patterns [42].

#### 2.2.9 Threat assessment and remediation analysis (TARA) methodology

TARA is an initiative of MITRE. It assesses cyberthreats and countermeasures. Cyberthreat susceptibility analysis (CTSA) entails a threat matrix representing an adversary's TTP. In conjunction with the CTSA, the cyber risk and remediation analysis (CRRA) is used to complete the TARA procedure [51]. CTSA requires determining which assets are in scope, identifying related TTPs, eliminating unlikely TTPs, utilising a ranking system, and constructing a threat matrix that displays the score, target assets, and type of attacker [42].

#### 2.2.10 Diamond model

Diamond is a model that formalizes scientific principles for intrusion analysis and links an attacker's skills to the target's infrastructure. It tracks attack groups that change targets and TTPs over time [52]. The term [52] originates from a diamond-shaped diagram depicting an intrusion's four components: an attacker, an infrastructure, an ability to act, and a victim. Like the ATT&CK and Kill Chain models, an attacker must use capability (TTP) rather than infrastructure against a target. In other words, it can correlate particular events and their connections, known as activity threads. The kill chain is then used to connect these activity threads. It is a way of doing intrusion analysis based on formal rules. It can also include factors such as phase, result, direction, methodology, and resources. It provides a way to find activity and link it to an attack using measures that can be tested and used repeatedly. Even though this approach is not very common, it is important to this study because it provides an effective formal way to model APTs [42].

### 2.3 APT detection mechanisms

APTs are designed to bypass controls such as firewalls, antivirus software, and IDSs, which protect against only known threats [53]. A defence-in-depth strategy is required to detect an APT attack at various levels and points in the network. Correlating events from different defence measures helps to protect an organization or entity from APT attacks. Generally, most detection methods used to detect APT are categorized into pattern-matching-based and anomaly-based detection [5].

#### 2.3.1 Pattern-matching-based detection

Pattern-matching-based detection is an outdated method used by orderly intrusion detection and prevention systems. Nevertheless, this method has its advantages. It is designed to discover malicious attacks and hacks by monitoring packets on a network and comparing network packets to a database of known attack patterns. Since APT actors use different stealthy and evasive methods, pattern-matching-based detection often fails. Therefore, the signature and pattern databases in pattern-matching IDSs must be continuously updated. Thus, high costs and false alarms are disadvantages of signature matching. As an example of this approach, Giura et al. [54] introduced the attack pyramid model as a way to identify APTs based on network events.

#### 2.3.2 Anomaly-based detection

An anomaly is the opposite of normal behaviour, and in this case, it refers to any suspicious behaviour that damages the system. It is also defined as unusual behaviours caused by interlopers who drop footprints in the computing environment [55]. The footprints are then compared to present data styles to discover anomalies and identify an unknown attack. Anomaly detection comprises the detection of doubtful network traffic, doubtful system activities, or clusters of irregular activities. A key characteristic of an APT attack is adapting a defender's approach to countering it. To defend against such a threat, one needs to recognize and adapt to the perpetrators' attempts. These methods should include collecting data from various sources, learning the data collected, and making predictions about the collected information to guess and respond to the next potential attack.

Anomaly detection methods can be classified into three classes according to the approaches used to learn about and identify the anomalies (supervised, unsupervised, and semi-supervised). The correct method for detecting anomalies depends on the labels available in the data set. In the supervised method, a classification algorithm needs a data set with ‘normal’ and ‘abnormal’ labels. This technique includes classifier training. The semi-supervised method uses a labelled training data set to model normal behaviour. Then, the user tests how probable the model is to generate each detected anomaly instance. Unsupervised anomaly detection relies solely on the core properties of unlabelled test data. As in most cases, the working assumption is that most data instances are normal. However, in the case of semi-supervised and unsupervised learning methods, false positives and negatives are problematic for detecting anomalies. This happens because normal and abnormal data are not distinguished. Further, user and system behaviour vary, requiring continuous learning and additional model updating.

For example, Siddiqui et al. [56] suggested a fractal-based anomaly classification algorithm to reduce the false-positive and false-negative ratios. Marchetti et al. [57] proposed a new framework named AUSPEX to assist human analysts in discovering and prioritizing weak signals regarding APT activities to combat APT-related threats. This was done after identifying the signature-based detection systems and antiviruses as ineffective against APTs. Moreover, Rubio et al. [58,59] have suggested using opinion dynamics algorithms to detect APT attacks, which can be used to model the spread of opinions in a population and identify the most affected areas within an industrial network. In Ref. [58], they suggested investigating APT detection in the context of topology modifications using a decision model of how a group of hierarchically selected nodes can function together. In addition, a response service was built that uses redundant links, secret sharing, and a dynamic routing protocol to respond to attacks of varying severity. In Ref. [59], they analysed the applicability of opinion dynamics to track an APT throughout its entire life cycle by correlating various anomalies over time and considering the persistence of threats and the importance of resources.

### 2.4 IDSs

Due to exponential network and application growth, the Open System Interconnection (OSI) model's random dynamic access relation is built on the fixed internet physical connection network, which has become more complex. Passive traffic collection and analysis help to manage networks and quickly identify security flaws. An IDS monitors traffic data to discover and prevent intrusions undermining an information system's confidentiality, integrity, and availability [60]. In addition, an IDS is a network security monitoring device or software that detects malicious activity or policy violations. Anderson's technical research report was the first to mention the concept of intrusion detection [14]. It presented a threat model that classifies threats as anomalies in user behaviour or the misuse of authorized access.

**Fig. 2:**
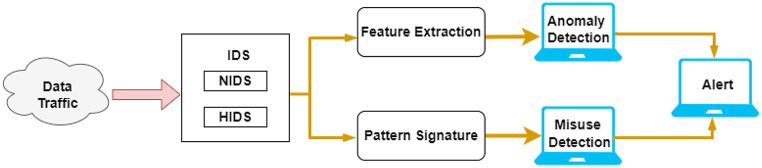
IDS operation.

Fig. 2 illustrates the three stages of an IDS's operation. The first stage is network- or host-based monitoring by sensors. This is followed by feature extraction or pattern recognition analysis. The last stage is the detection of the anomaly or intrusion. The IDS intercepts and analyses a system's data traffic to detect potentially harmful activities [60].

An IDS's goal is quickly identifying malware, which a traditional firewall cannot do [61]. IDS architecture can be fundamentally divided into host IDS (HIDS) and network IDS (NIDS). Host-based detection fundamentally matches the process of the data record of a single host. It protects a single system against snooping or malicious attacks that damage the operating system or files [14]. This obviously does not meet network security requirements. Thus, network-based detection is built by adding protocol information and traffic to the host-based detection. NIDS monitors network traffic and protocol data to discover intrusions. The NIDS can be hardware or software-based [60].

Technically, there are two types of IDSs: abnormal and misuse intrusion detection [62]. In abnormal intrusion detection, the rule set of abnormal behaviour detection is the normal system operation mode [14]. An alarm signal is generated when there is a deviation from the normal system. This method can record exploratory behaviour and the prescribed ‘normal’ action. However, the false-alarm rate will be higher because the system's normal mode is dynamic and cannot be fully normalized when establishing a detection system.

In contrast, misuse intrusion detection is a model of harmful system behaviour. It generates an alarm when it detects behaviour that matches the dangerous pattern. This method is accurate for clear matching, particularly for the known attack paradigm. However, there is a high missing-report rate because it is nearly impossible to passively summarize the entire sample of harmful behaviour in the presence of numerous aggressive behaviours [14].

Despite extensive study of IDSs, several fundamental issues persist. IDSs must be extra precise, with fewer false alarms, and face other challenges [62]. Therefore, network security situational awareness (NSSA) is used to improve this approach. NSSA assists network safety personnel in comprehending the entire network's security status, identifying problems and abnormal activities on the current network, and providing the corresponding feedback for network enhancement. NSSA is a security concept that can perceive the network danger from a universal perspective and analyse the intentions of attackers with the help of a network security management system [63]. It provides an important basis for management decision-making.

NSSA includes three aspects: extraction of elements of a network security situation, assessment of a situation of network security, and prediction of the network security situation. Table 4 presents the differences between IDSs and NSSA based on the range of information collected from the network, function, analysis yielded, detection time, and detection efficiency.

**Table 4:** The difference between intrusion detection systems (IDSs) and network security situational awareness (NSSA).

IDSs	NSSA
Focus on the presence or occurrence of attacks (anomaly events)	Concerned with a network's overall security
Collect data on network core elements	Requires information collection of all network elements
Main function: detection of abnormal/misuse cases	Core function: prediction of the security situation
Can perform behaviour analysis, which is part of fusion analysis	Can perform fusion analysis and decision support
Alarm sounds after the attack occurs; thus, real-time network security is difficult to ensure	Detection and alarm occur before the attack to keep the network safe
Detection efficiency is high rate of false alarms and low rate of real-time responses	Detection of large data in real time based on flow data improves timeliness; data fusion on multiple levels produces overall perception

## 3 Research methodology

We followed Budgen and Brereton's SLR guidelines [64], which confirm the identification, evaluation, and interpretation of all available research pertinent to a research question or topic. This approach also contributes to minimizing the risk of bias in publication and enables researchers to identify new research avenues for future reviews.

**Fig. 3:**
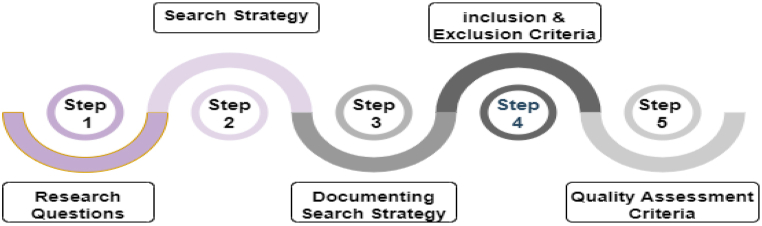
Steps of systematic literature review.

An SLR was conducted to answer the questions identified as to whether those topics might arise in a realistic context. A comprehensive review was undertaken based on the research collection, and the most important studies that addressed the problems were documented. This SLR was entirely concerned with acquiring the most important papers, which it treated as primary sources to obtain the best outcomes. These papers were evaluated. The steps of our SLR are shown in Fig. 3.

The sequence of steps in the SLR protocol identified the research questions and strategy formulation. It also determined the search strategy, inclusion-exclusion criteria, and evaluation of quality criteria. All these considerations are addressed in this section.

### 3.1 Research questions

This section outlines the research questions used to classify the prime studies, forming a key part of this study. Table 5 presents three research questions associated with APT detection mechanisms and provides detailed answers.

**Table 5:** Research questions.

No. of question	Research question	Description
RQ1	What Is the Current State of the Art Concerning Detection Models for APT on Smartphones?	This question enabled us to explore the common characteristics of detection models and organize them within a specific taxonomy. Furthermore, this taxonomy was used to consider the available capabilities that contribute to detecting APT attacks.
RQ2	What machine learning (ML) techniques have been applied to detect APTs?	Identify the categories of ML techniques applied in APT detection.
RQ3	What situational awareness (SA) models in cybersecurity were used in previous studies?	This question enabled us to identify different SA models used in cybersecurity.

### 3.2 Search strategy

The authors used related search terms to find studies related to APT detection mechanisms. Boolean expressions such as ‘AND’ and ‘OR’ were used to combine search terms. Thus, an independent group of experts chose the following search string: (‘advanced persistent threat*‘) AND (mobile OR smartphone OR computer OR IoT OR ‘cloud computing’ OR ‘Situation* Awareness’).

An SLR needs to use more than one database for a complete science mapping analysis to find results. We used six online databases: Digital Library of IEEE Xplore, ScienceDirect, Digital Library of ACM, Scopus, Springer Link, and Web of Science.

### 3.3 Documenting search strategy

**Fig. 4:**
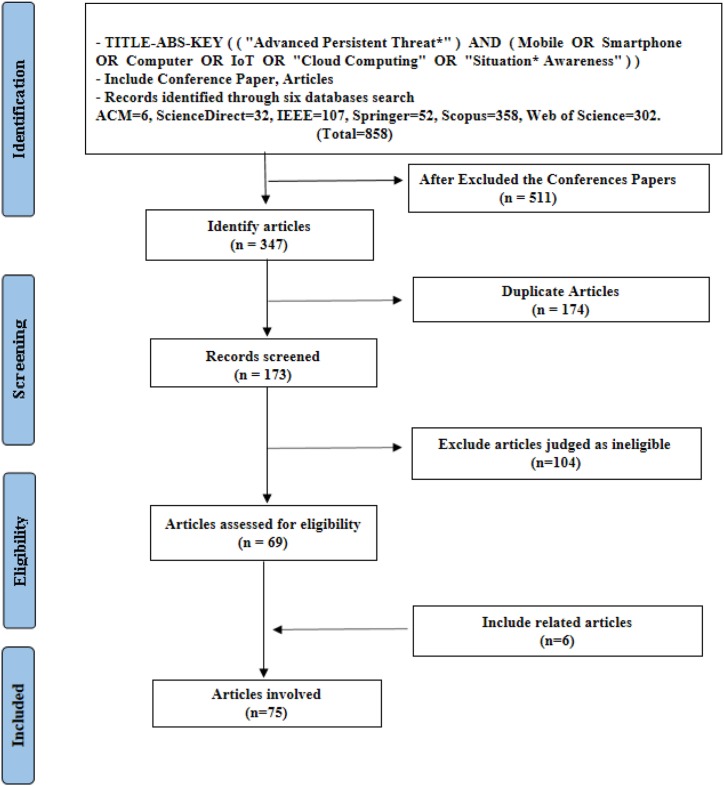
PRISMA flow diagram showing research strategy protocol.

Fig. 4 shows the flow of the search strategy. Conference papers were first excluded from the search results, and then duplicate articles were eliminated. Furthermore, the included and excluded papers were documented in a list.

### 3.4 Inclusion and exclusion criteria

The authors searched six electronic databases for related studies using a set of inclusion and exclusion criteria to ensure the selected studies met the objectives of the SLR, as illustrated in Table 6.

**Table 6:** Study inclusion and exclusion criteria.

Inclusion criteria	Exclusion criteria
Studies that were published in English	Studies published in languages other than English
Search keywords appear in the title, abstract, or article keywords	Duplicate research papers
Studies that present advanced persistent threat detection mechanisms	Studies that do not address the research questions or sufficiently identify the subject.
Studies published in journals during the period 2012–2022	Studies that have fewer than 6 pages

### 3.5 Quality assessment criteria

We screened the selected studies and assessed their quality using the quality assessment criteria in Table 7. To ensure the results were reliable, we cross-checked the studies chosen. The final data set included 69 studies and six studies related to situational awareness (SA) models in cybersecurity.

**Table 7:** Criteria of quality assessment.

Id	Quality Criteria
1	Are the study's objectives clear?
2	Does the study discuss the advanced persistent threat detection mechanism clearly?
3	Is the detection model clearly stated?
4	Are the performance metrics clear?
5	Does the study add to this systematic literature review?

## 4 Analysis and findings of research questions

This section aims to analyse primary studies and show the obtained results. The authors describe primary studies and present the SLR results that align with the research questions.

### 4.1 Description of studies

This section summarizes 75 studies by publication date and source.

#### 4.1.1 Publication time

**Fig. 5:**
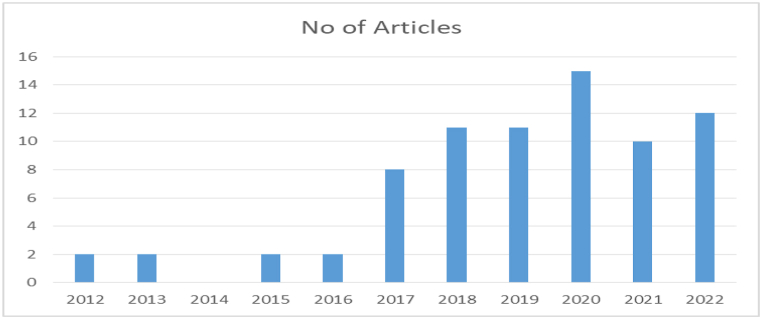
Distribution of articles based on year of publication.

The number of studies published from 2012 to 2022, by year, is 2, 2, 0, 2, 2, 8, 11, 11, 15, 10, and 12, respectively, as shown in Fig. 5. Accordingly, most studies were published in 2020, while none were published in 2014. We collected data before the end of 2022; thus, the 2022 papers were published between January and September. The number of studies done on the detection mechanisms of APT grew substantially from 2017 to 2021.

#### 4.1.2 Publication source

Six online databases, ACM, IEEE Xplore, Scopus, ScienceDirect, Springer, and Web of Science, were consulted as literature sources. These databases were chosen due to their scholarly rigor and coverage of our discussion area. In addition, we modified search result references and used standard internet search engines such as Google Scholar as a secondary source to ensure and validate that research has been included within those six databases. Table 8 shows all the articles included in this study, which were published across 42 journals. This table includes the name of each journal and the number of primary articles published in it. Furthermore, it presents *IEEE Access*, *Future Generation Computer Systems*, *Computers & Security*, and *Journal of Intelligent & Fuzzy Systems* as the first four publication sources. The published papers are 8, 5, 5, and 5, respectively. Table 8 shows that the articles published in these three journals represent about 29% of the articles included in this study.

**Table 8:** Results of the publication source.

ID	Journal name	No. of articles
1	IEEE Access	8
2	Future Generation Computer Systems	5
3	Computers & Security	5
4	Journal of Intelligent & Fuzzy Systems	5
5	IEEE Transactions on Dependable & Secure Computing	4
6	The Journal of Supercomputing	3
7	Electronics	3
8	IEEE Internet of Things	3
9	Computer Networks	2
10	Neural Computing and Applications	2
11	Cluster Computing	2
12	Security and Communication Networks	2
13	Applied Sciences	2
14	Journal of Wireless Mobile Networks, Ubiquitous Computing, and Dependable Applications	1
15	IEEE Transactions on Mobile Computing	1
16	Procedia Computer Science	1
17	IEEE Network	1
18	IEEE Transactions on Industrial Informatics	1
19	IEEE Systems	1
20	IEEE Transactions on Information Forensics & Security	1
21	ACM Transactions on Information & System Security	1
22	Neurocomputing	1
23	Information Sciences	1
24	Computers, Materials & Continua	1
25	International Journal of Intelligent Information Technologies	1
26	HAL Open Science	1
27	KSII Transactions on Internet and Information Systems	1
28	Computer Virology & Hacking Techniques	1
29	Ambient Intelligence & Humanized Computing	1
30	Journal of Big Data	1
31	Neural Processing Letters	1
32	EURASIP Journal on Information Security	1
33	Statistical Analysis & Data Mining: The ASA Data Science Journal	1
34	International Journal of Advanced Computer Science & Applications	1
35	Mathematical Problems in Engineering	1
36	Concurrency and Computation: Practice and Experience	1
37	Multimedia Tools and Applications	1
38	Wireless Communications and Mobile Computing	1
39	Egyptian Informatics Journal	1
40	Applied Intelligence	1
41	Journal of Computer Security	1
42	Array	1

### 4.2 RQ1: what is the current state of the art concerning detection models for APT on smartphones?

**Fig. 6:**
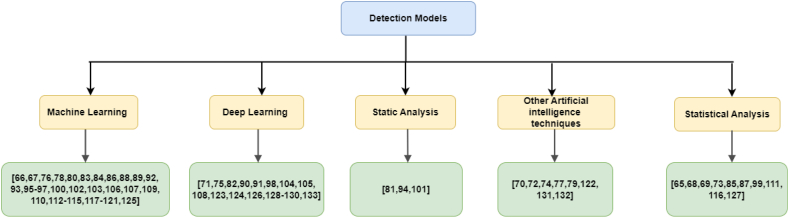
Classification of detection methods.

This section presents several studies that deal with detection methods or models used to detect an APT attack, and that would be classified as such based on the NIST guidelines [45]. These studies totalled 69. Detection methods were classified as based on either ML, deep learning, statistical analysis, static analysis, or other artificial intelligence (AI) techniques, as shown in Fig. 6.

#### 4.2.1 ML–based APT detection methods

**Fig. 7:**
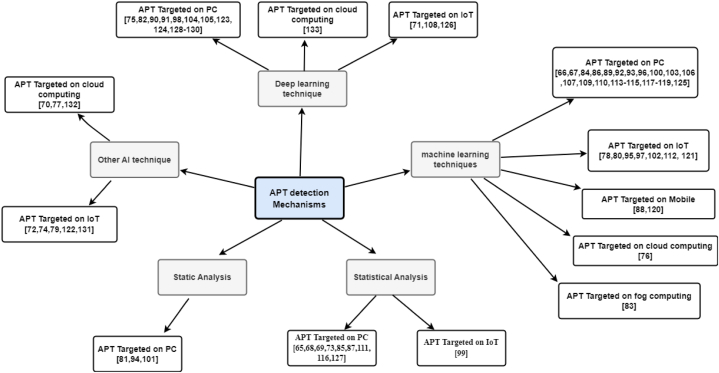
Taxonomy of APT detection mechanisms based on the platform.

ML is a subgroup of AI [123]. ML is divided into four main categories based on the model's construction [133]: (i) supervised learning, where the data set contains the labelled predictor features; (ii) unsupervised learning, where the data set comprises the predictor features without the labels; (iii) semi-supervised learning, where the data set comprises the predictor features, some of which have labels and some of which do not; and (iv) reinforcement learning, which enables software agents and machines to automatically choose the best course of action in a given situation. This learning method is based on reward or penalty, and its ultimate goal is to use the insights of actors within the environment to take action to increase the reward or reduce the risk [134]. ML aims to create algorithms that can learn from the past and improve the system over time. By supplying the algorithms with information, the systems can change their internal programming to improve at a particular task. ML can assist system administrators in finding suspicious behaviour, such as an APT, in an enterprise network, and ML techniques are commonly used to detect APT attacks (see RQ2). Fig. 7 explains the different ML algorithms used to detect APT attacks based on the platform; of these, approximately 70% targeted the computer, while the remainder targeted the internet of things (IoT), cloud computing, and mobile devices.

#### 4.2.2 Deep Learning–Based APT detection methods

**Fig. 8:**
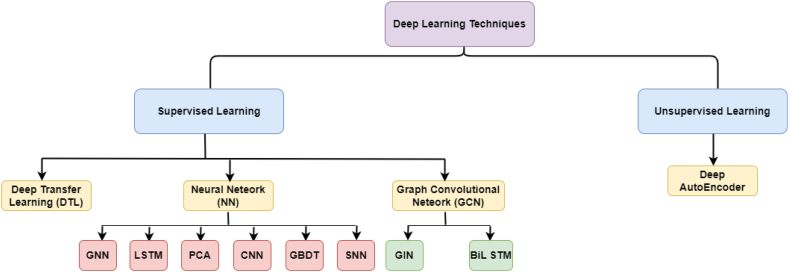
Taxonomy of APT detection based on deep learning techniques.

Deep learning is a subset of AI-derived from biological neural networks in the human brain [75]. Pulses or electrical signals carry information and data into and out of nerve cells and neurons. Deep learning uses multilayered deep neural networks, which learn features in layers. A deep learning network is called a neural network with more than two hidden layers. Deep networks outperform other ML models such as decision trees, Bayesian networks, and support vector machines (SVMs) on unstructured data, and they have higher accuracy than those models, but they require a lot of training data and appropriate hardware and software. Consequently, they are less commonly used for APT detection when compared with ML. Thus, many studies adopt deep learning to detect APT. Fig. 8 explains the different deep learning algorithms that were used to detect APT attacks.

For example, three studies [82,104,108] used the deep autoencoder algorithm. Rahman et al. [71] proposed using deep transfer learning (DTL) to improve IoT-aligned Industry 4.0 security. Joloudari et al. [75] used a six-layer model of deep learning and achieved better accuracy and false-positive rate performance. Shang et al. [90] proposed a study combining convolutional neural network (CNN), principal component analysis (PCA), and gradient-boosted decision tree (GBDT) algorithms to detect unknown malicious samples, and the results demonstrated high performance. Fang et al. [91] proposed the LMTracker method, which uses heterogeneous graphs. The results of path-level detection can immediately be used to track down attack activities and fix network weaknesses. Xuan et al. [98] proposed the BiLSTM-GCN model, which demonstrated the best performance of the models tested in those studies. Although applying deep learning to flow network analysis to discover APT attacks is a good idea, Xuan and Dao also suggested the neural network–long short-term memory (NN-LSTM) model that significantly improves APT IP detection [105].

Bodström and Hämäläinen [123] proposed a model-based theoretic approach or idea related to APT attacks, stating that APT attacks are persistent, multistage attacks that use the whole network flow as input. For that, experiments show that a deep learning stack that uses sequential neural networks provides the best and most flexible architecture for detecting APT attacks. Xuan and Huong [124] proposed a method to analyse and evaluate behaviour profiles using the Graph Isomorphism Network (GIN) to improve the efficiency of analysing and detecting APT malware on workstations. In addition, Xuan et al. [129] proposed a new method for analysing and detecting APT malware on endpoint devices, such as unauthorised intrusions and insiders, using CNN-attention, a combination of CNN and the attention network. Further, Xuan and Duong [130] proposed a model that improves analysis efficiency and APT malware detection based on network traffic using CNN-LSTM and the attention network. In addition, Xuan et al. [128] devised a new method for using LSTM and bidirectional LSTM algorithms to synthesize and analyse the peculiar behaviour of APT malware on workstations and to detect it.

Niu et al. [126] devised a way to classify malware that uses association rules and time sequence features, and they used an improved LSTM model to find malware. Abdullayeva [133] suggested a method for detecting APT attacks in cloud computing using an autoencoder and softmax regression algorithm. The method involves training an autoencoder to reconstruct normal network traffic and then using softmax regression to classify incoming traffic as normal or malicious.

(CNN: convolutional neural network, PCA: principal component analysis, GBDT: gradient-boosted decision tree, LSTM: long short-term memory, SNN: sequential neural network, GNN: graph neural network, GIN: graph isomorphism network, BiLSTM: bidirectional long short-term memory).

#### 4.2.3 Static Analysis–Based APT detection methods

Static analysis is a form of code analysis method that receives a software package's origin code or binary code as input [138] and then inspects the code without running the software package to ensure its security and reliability. When compared to dynamic analysis, static analysis does not need to execute the application, so it is efficient and fast. As a result, static analysis is widely employed for software traceability and anomaly detection, such as in identifying APT. For example, Dube et al. [81] used static heuristic features to extend the target recognition architecture of performance-based malware. Santos et al. [94] suggested using opcode frequency to detect unknown malware families. Chakkaravarthy et al. [101] used static and dynamic analysis to detect the APTs in memory during its execution.

#### 4.2.4 Other AI-based APT detection methods

AI makes machines function similarly to humans in solving complex problems [136]. It involves areas such as natural language processing and the environment and takes actions that increase people's chances to achieve their objectives. Many studies have adopted AI to identify attacks compromising the security of robotics, computer vision, information retrieval, ML, and deep learning. AI uses agents that focus on these applications and systems in an organization. For example, several studies [70,77,132] used game theory strategies to improve the detection model and guide defence strategies. Ghafri et al. [58] used BotDet detection to prove its real-time detection capability. Ma et al. [74] proposed a method for detecting C&C domain names based on the domain name map structure, which allowed them to discover C&C domain names even with a small initial domain name being used. Khan et al. [79] used electromagnetic radiation to detect malware. Cheng et al. [122] suggested a new approach for perceiving the cyber situation of IoT systems based on recognising zero-day attack action within APT (CSPAPTM). Rubio et al. [131] suggested that opinion dynamics could track an APT throughout its entire life cycle by correlating various anomalies over time and accounting for the persistence of threats and the importance of resources.

#### 4.2.5 Statistical Analysis–Based APT detection methods

**Fig. 9:**
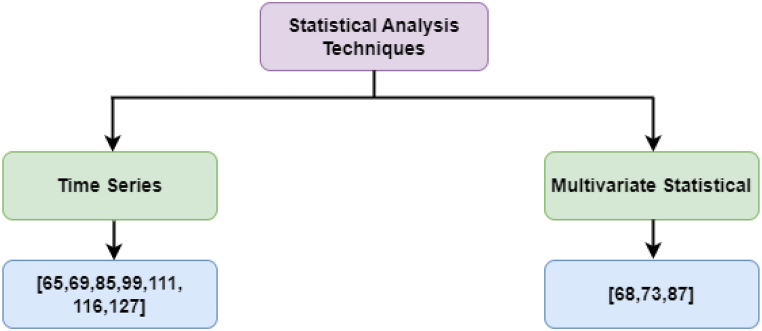
Statistical analysis techniques.

Statistical analysis involves gathering, exploring, and using huge amounts of data to determine fundamental styles and tendencies [137]. It is applied daily in research, manufacturing, and government and has become the scientific basis on which decisions in those industries are sometimes made. In cybersecurity, comparatively basic statistical data processing methods are used to extract properties from data samples. This approach is typically used in anomaly detection and data pre-processing. The core advantage of this approach is that it is simple and does not need large data sets. However, it does not handle multidimensional data well, and evaluation decisions require prior knowledge. Fig. 9 shows articles that used statistical analysis techniques to detect APT attacks.

For example, Yan et al. [65] proposed using structured intrusion detection to detect APT. Their method uses high-level structured data captured in a network traffic time series. Ioannou et al. [68] devised a model to address exfiltration APT attacks called the Markov Multi-stage Transferable Belief Model, in which the Kill Chain model and the attack tree were combined to provide multistage attack situation awareness. Khosravi and Ladani [69] proposed a real-time method for detecting APT-based cyberattacks based on causal analysis of security and nonsecurity sensor alerts. The proposed method uses dynamic programming to analyse alerts from each host alone and conducts a long-term analysis of the attack process. Xiong et al. [73] proposed CONAN to detect APT attacks using a state transition approach such as finite state automata (FSA). Friedberg et al. [87] suggested a method for detecting anomalies caused by APTs, such as providing direct access to database servers and copying huge amounts of data. This method detects anomalies by combining several rules that guide the model. The proposed approach performed well on the supervisory control and data acquisition (SCADA) data set. The proposed approach is also expected to work well on real data.

Cheng et al. [99] developed the APT Alerts and Logs Correlation Method (APTALCM) to help IoT systems understand the cyber situation. They provided a framework for using APTALCM on IoT systems based on edge computing. Lajevardi and Amini [111] proposed an approach to discover slow and low-level APTs. It relies on a knowledge-based semantic correlation engine. Where Vermiform windows are used in this proposed approach, it has two phases: expanding and shrinking. In expanding, the authors used SANSA and big data frameworks such as Spark to link large events, whilst shrinking reduces the sliding window's events, and they were done to enhance the prior solution proposed in Ref. [85] to detect slow APTs, apart from low-level and hybrid APTs.

Based on the detection methods or models used to detect an APT attack in this study, we have classified detection methods into five categories: ML-, deep learning–, static analysis–, other AI-, and statistical analysis–based detection methods. ML-based detection methods are the most commonly used in APT detection. However, Table 9 illustrates that the metric we relied on to prefer ML techniques over other techniques is the APT fingerprint, or APT TTP, which is the outcome of our proposed model in this study. In light of this, we found some studies that applied this, most of which used ML.

**Table 9:** Summary of studies on advanced persistent threat (APT) detection techniques, aggregated by single-stage or multistage APT.

Technique	APT detection (single stage)	APT fingerprint (multistage)
Machine learning	[66,67,76,78,80,83,84,86,88,92,100,102,103,107,109,110,113,114,[117], [118], [119], [120]]	[89,93,[95], [96], [97],^112^,^121^,^125^]
Deep learning	[71,75,82,90,91,98,104,105,108,123,126,129,130,133]	[124,128]
Static analysis	[81,94,101]	
Other artificial intelligence techniques	[70,72,74,77,79,122,132]	[131]
Statistical analysis	[65,68,73,85,87,116]	[69,99,111,127]

### 4.3 RQ2: what ML techniques have been applied to detect APTs?

**Fig. 10:**
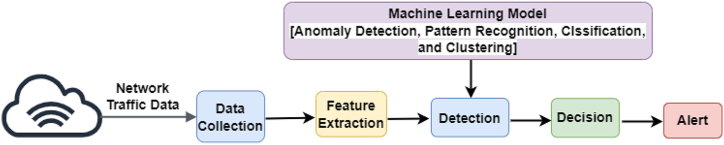
Steps of cyberattack detection.

In answer to RQ2, we analyse and summarize experiential evidence in detail. Fig. 10 demonstrates the steps of the process of experiential experiments in cyberattack (e.g., APT) detection.

The first step, data collection, is about the amount of data collected from network traffic. The cybersecurity detection engine (e.g., IDS) obtains all related input data; for instance, network traffic data. Obtaining data to create malware detection schemes (e.g., for APT) is important. The purpose of data gathering is to gather benign and malicious data sets. Generally, the more empirical the data sets, the more effective the outcomes are. In the second step, after data collection, the cybersecurity detection engine extracts the features it desires (e.g., IP addresses of network data); this is called feature extraction, a process that categorizes significant attributes or features of the data. Features are either static or dynamic. Once the features are extracted, feature reduction methods are adopted to limit and select the important features.

The third step, detection, aims to find a suitable model to differentiate or detect malicious packets from benign packets by choosing an appropriate ML model to build the model and classify packets as normal or as malware. ML methods used to detect APT can be categorized into anomaly detection, pattern recognition, classification, or clustering of the data for training [139]. Finally, after detecting malicious packets, the cyber defence engine informs the network administration by sending an alert or blocking those packets to keep the network safe.

The ML technique depends on splitting the data set into training, validation, and test sets. Training is used to appropriate the parameters and train the model; validation is used to predict observation responses and evaluate a model suitable for the training data set while changing hyperparameters and testing sets that offer an unbiased assessment of the final model suitable for the training data set. Numerous metrics, including, for instance, precision, recall, accuracy, and F1-score, are used to measure the performance of the executed method.

**Fig. 11:**
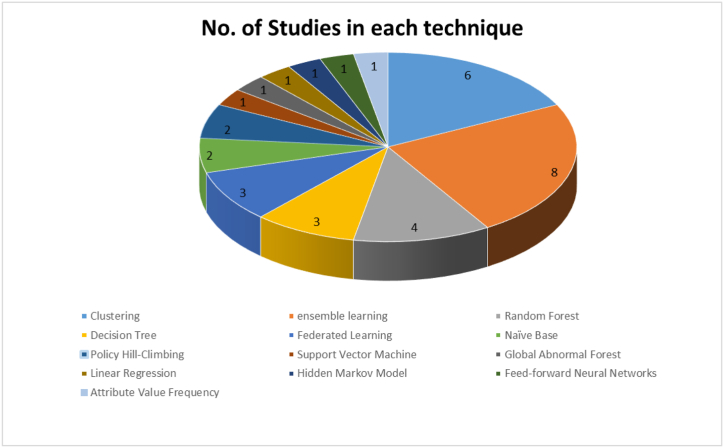
Distribution of machine learning techniques.

This study introduced 34 articles on how to detect APT attacks using ML techniques. These studies used different ML techniques, and some used more than one technique in APT attack detection. Fig. 11 shows how the ML techniques used in this study are split up.

We discuss the 13 techniques identified during the review in the subsections below.

#### 4.3.1 Clustering technique

The clustering technique is an unsupervised learning method (k-means, fuzzy c-means, and hierarchical) used to develop predictive models. Unsupervised ML models can detect and classify spam. Many primary studies employed clustering techniques to detect APT attacks. For example, Cho and Nam [67] proposed an approach that relies on monitoring access to unknown domains. Once detected, warning signals are created for the user. Two sets of authors [107,118] focused on the computer device and used fuzzy means algorithms to categorize the malware analysis approaches into those based on static analysis and those based on dynamic analysis. Three articles [86,93,106] focused on the computer device. They intended to address the early detection of APTs in big and constantly changing network systems.

#### 4.3.2 Ensemble learning

Ensemble learning aims to integrate ML algorithms seamlessly. Thus, each algorithm's complementary information improves the overall model's performance and achieves better performance than any single algorithm alone [140]. Ensemble learning can be combined with different ML models for classification, clustering, and so on. Existing ensemble learning methods can be categorized as supervised, semi-supervised, and clustering. For example, Moustafa et al. [83] proposed the outlier Dirichlet mixture–based anomaly detection system (ODM-ADS), a statistical method for finding anomalies that uses adversarial learning. It outperformed seven peer algorithms in discovering network traffic abnormalities and zero-day attacks via learning patterns from normal and abnormal traffic through the training phase. Ghafir et al. [89] introduced an approach to identify and predict APTs called MLAPT that relies on different ML algorithms. To secure the industrial IoT (IIoT), Javed et al. [97] presented the APT intelligent detection and classification system.

Where a comparative analysis of ML techniques was conducted, the results indicated that the AdaBoost classifier outperforms the others with an accuracy rate of 99.9% and an execution time of 0.012 s. Chu et al. [100] found the SVM algorithm to have a detection accuracy rate of 97.22% and a radial basis function (RBF) that has the best performance compared to other classification algorithms such as J48 decision tree, multilayer perception (MLP), and naïve Bayes. Alqahtani et al. [102] proposed an approach to detect IoT botnet attacks by determining the most relevant features; it builds on a Fisher-score-based feature-selection method and the GXGBoost model. To detect APT attacks through network traffic in a distributed environment, Sharma et al. [112] proposed the distributed framework architecture for APTs detection (DFA-AD) approach, which examines the relationship between events generated by different classifiers. After examining the results produced by the event association module, the voting process starts issuing an alert about the APT attack.

By analysing the mobile DNS record, Niu et al. [120] proposed a method for detecting C&C domains with high accuracy using malicious APT code. The domain was registered using Alexa and VirusTotal, and the C&C malware domain was found using the geographical adaptive fidelity (GAF) algorithm, which was more accurate than the local outlier factor (LOF), k-nearest neighbours (K-NN), and isolation forest algorithms by more than 99%. Finally, Al-Saraireh and Masarweh [125] developed a model that uses extreme gradient boosting and the study of variance feature selection to find APT attacks at different stages. This model was more accurate than the random forest (RF), K-NN, and decision tree algorithms, scoring 99.89%.

#### 4.3.3 RF algorithm

The RF algorithm is a popular supervised learning algorithm for classification and regression. An RF classifier is an ensemble classifier that uses a randomly selected subset of training samples and variables to generate multiple decision trees. In feature selection, the RF algorithm was used to reduce the dimensions of the data set to the most significant features. The RF algorithm is widely used in detecting APT attacks in preliminary studies. The authors in Refs. [80,96,117,119] focused on detecting APT attacks on computer devices. Niu et al. [80] proposed a trained RF model to detect APT malware domain names based on DNS traffic from unmanned aerial vehicles (UAVs). The proposed detection method achieved 94% accuracy in experiments. Xuan et al. [96] developed an approach for APT attack discovery built on multilayer analysis through computation and network traffic analysis to discover and synthesize abnormal symbols and behaviours. Bolton and Anderson-Cook [117] presented a three-stage approach to classifying new malware into a family by comparing their similarity with existing persistent traces and allocating them to the most similar family. Cho et al. [119] suggested using the RF algorithm to detect how APT attacks rely on C&C servers. Also listed were Symantec, McAfee, Kaspersky Lab, Forcepoint, Palo Alto Networks, Fortinet, Cisco, and FireEye as APT attack detection tools.

#### 4.3.4 Decision tree

A decision tree is one of the classification techniques for ML that relies on a divide-and-conquer strategy. Its models are precise, steady, and easy to interpret. It is constructed based on tree-like decision rules. The models comprise nodes and leaves, where the nodes are individual features, and the leaves are the class labels. These models can help solve nonlinear problems. Some primary studies use decision tree classification, which requires extensive storage capacity, to detect APT attacks. Zhao et al. [66] suggested a system to detect APT malware infections. The system is divided into two stages: detecting malicious C&C domains and analysing related internet protocols for questionable and malicious traffic. To find malicious DNSs, the authors used a J48 decision tree algorithm, signature-based detection, and anomaly-based detection. To detect APT attacks that change in their mechanics or something of the sort after the intrusion, Moon et al. [113] suggested a decision tree–based IDS. They also proposed a malware detection approach that relies on process behaviour [114]. Their proposal overcomes the limitations of signature-based IDSs.

#### 4.3.5 Federated learning

Federated learning (FL), an ML algorithm, trains an algorithm on several edge devices or servers that are not connected without sharing data samples. Some primary studies used FL to detect APT attacks. Xu et al. [76] showed that robust edge intelligence could achieve high-accuracy detection and good computational performance. FL was used by Taheri et al. [78] to develop an FL-based architecture (Fed-IIoT) for finding Android malware in the IIoT. Cheng et al. [121] created the APT Prediction Method based on Differentially Private Federated Learning (APTPMFL), which is an APT prediction method for the 5G-enabled IoT based on FL.

#### 4.3.6 Bayesian algorithms, specifically Naïve Bayes

Bayesian algorithms, naïve Bayes in particular, are well known for being easy to use, requiring little training, and being fast. The naïve Bayes algorithm is based on Bayes’ theorem, with a strong assumption that all the predictors are independent of each other. In some primary studies, naïve Bayes was used to detect APT attacks. Ahmed et al. [95] matched Cyber Kill Chain alerts to identify APT attacks. Feature selection is used to improve APT prediction accuracy. An approach of APT-Dt-KC adapts the Cyber Kill Chain model to identify fuzzy APT attack features, which can help to detect APT attacks. This approach was proposed by Panahnejad and Mirabi [110].

#### 4.3.7 Policy hill-climbing

The policy hill-climbing (PHC) algorithm is a reinforcement learning algorithm that aims to find the optimal policy by iteratively improving the current policy through small changes and evaluating the resulting changes in performance [135]. In addition, some primary studies used the PHC with game theory to improve the detection of APT attacks; Xiao et al. [70] proposed a PHC-based detection scheme to enhance policy unpredictability and deceive the attacker in a dynamic game. A ‘hot-booting’ technique was devised to accelerate the learning speed of PHC-based detection by using experiences in similar scenarios to initialize the quality values. Simulation results demonstrate that the proposed strategy can enhance detection performance with more data protection and cloud utilities in an attacker's presence compared to a conventional Q-learning strategy. Min et al. [132] presented a CPU-allocation strategy based on ‘hot-booting’ PHC that uses comparable scenarios to set quality values to speed up learning. Simulations reveal that reinforcement learning–based CPU allocation can increase cloud storage system data safety and utility compared to Q-learning-based CPU allocation against APTs.

#### 4.3.8 Attribute value frequency

Attribute value frequency (AVF) is one type of unsupervised categorical anomaly detection. It is a simple and quick way to find outliers in categorical data. It reduces the necessary number of data scans because it does not need to create or search through different attribute values or item sets. Berrada et al. [84] took Boolean-valued features from the provenance graph, which they called contexts, and used unsupervised learning techniques to treat cyberattack detection as an anomaly detection task.

#### 4.3.9 Global abnormal forest

Global abnormal forest (GAF) is a supervised ML approach. Some of the primary studies used the GAF algorithm to detect APT attacks. In a study by Xiang et al. [88], an approach was proposed using an ML algorithm to analyse DNS logs for detecting APT attacks on mobile devices. The authors extracted different features from two platforms, depending on the device (i.e., PC and mobile platforms).

#### 4.3.10 Linear regression

Linear regression (LR) attempts to model the association between two variables by a suitable linear equation based on the observed data, where one variable is an explanatory variable and the other a dependent variable. Regression analysis in cybersecurity answers questions about a response variable's dependencies. Reducing the dependent variable (security threats) depends on the independent variable (network security tools). Some primary studies used LR to detect APT attacks. Burnap et al. [92] compared legitimate and malicious software using machine activity metrics and a self-organizing feature map. The APT detection method showed promise.

#### 4.3.11 Hidden markov model

The hidden Markov model (HMM) is a statistical model that is also used in ML. It can be used to explain how things change over time when they depend on internal factors that cannot be seen. Brogi [103] proposed real-time APT detection using an HMM.

#### 4.3.12 SVM

The SVM is a way to learn with supervision and is used for regression and classification. The SVM puts vectors that are fed into it into a space with many dimensions. They can work well in both binary and multiclass situations. Some of the primary studies that looked for APT attacks used SVM. Wang et al. [109] developed a multi-feature SVM algorithm to detect APT attacks.

#### 4.3.13 Extreme learning machines (ELM)

Extreme learning machines (ELMs) are unsupervised learning techniques. It is a feed-forward neural network for classification, regression, clustering, sparse approximation, compression, and learning features. It can have one or more layers of hidden nodes. Shi et al. [115] formulated a way to use extreme ML to find bad domain names. This method uses ELMs to describe a domain name's construction-based, IP-based, TTL-based, and Whois-based features.

Overall, Ensemble Learning makes up the biggest share of the most common ML models for APT detection in this study.

### 4.4 RQ3: what SA models in cybersecurity were used in previous studies?

**Fig. 12:**
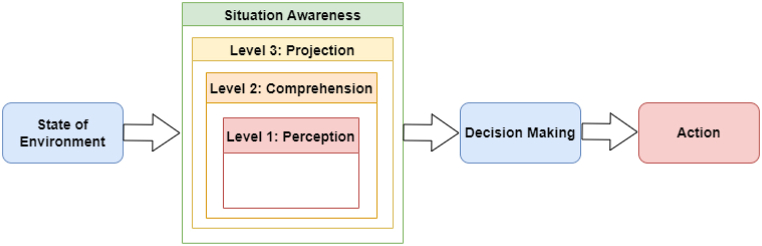
Endsley's situational awareness model.

Cybersecurity is important in our highly networked society, and SA is important in cyber defence. Understanding cyberspace events and entities involves science, technology, and practice. ‘Situational awareness’ has several meanings, and although the term was first defined in the mid-1980s, its use dates to World War I [141]. Until 1995, nearly all definitions of SA were military-oriented, reflecting the growing interest in pilot awareness during flight; in recent years, it has been used in cybersecurity. SA is based on a three-layered model (Endsley's model) in which the levels are perception, comprehension, and projection, as illustrated in Fig. 12.

‘Cyber situational awareness’ (CSA) in the context of cybersecurity means predicting and accurately responding to possible cyberthreats against a system or network [142]. It has three important parts [142]:

(1) Situation recognition (also known as situation perception) is concerned with determining the incidence of an attack and the attack's type, source, and target. This aspect focuses on the data and information gathered's accuracy, completeness, and timeliness.
(2) Situation comprehension includes assessing the attack's impact (damage assessment) for both present and future impacts. Also, it includes being aware of the attacker's behaviour, including the attack's trends and intent. One needs to comprehend the situation to determine what caused the current situation.
(3) Situation projection means being aware of how the situation changes and what other effects it might have. A well-thought-out design for a system's SA would help decision-makers to understand what is going on and how secure the system is. Once a decision is made, the response actions are planned and executed.

However, higher situation awareness levels, from perception to projection, are still manual, time-consuming, and error-prone. There are still not enough situation awareness systems that can respond to a changing environment by being able to change independently without a lot of help from people or outside agents.

The authors surveyed the studies that discussed situation awareness models in the field of cybersecurity, and these studies were classified according to the type of model and the mechanism used against cyberthreats and the target platform, as shown in Table 10.

**Table 10:** Categories of situational awareness in cyberattacks used in the studies reviewed.

Paper	SAM					Defence mechanism					Platform				Type of attack	
	SA	OODA	CSA	ECSA	JDL-DF	Detection	Prevention	Prediction	Mitigation	Identification	Computer	Mobile	Cloud computing	IoT	Traditional attack	APT attack
[68]																
[99]																
[121]																
[122]																
[143]																
[144]																
[145]																
[146]																
[147]																
[148]																

SAM: situational awareness model; OODA: observe, orient, decide, act model; CSA: cyber situational awareness model; ECSA: effective cyber situational awareness model; JDL-DF: Joint Directors of Laboratories data fusion model.

#### 4.4.1 Endsley's SA model

Endsley's SA model involves perception, comprehension, and projection. The perception of environmental elements is the first step toward SA. This level covers the state, properties, and dynamics of environmental elements. Understanding the current state is based on Level 1 outputs, while projecting the future state involves predicting what environmental elements will do. People who know how things are and how they are changing do this to determine what is happening. Xu et al. [143] developed a semantic-ontology-based method for situation reasoning that provides a complete picture of the security situation and makes it easier to respond to emergencies.

Two articles [144,145] discussed network threats discovered in cloud computing. Edge and fog system installations uncover potential security threats that can be countered with a conceptual framework proposed by Rapuzzi and Repetto [144]. There are three layers to the conceptual framework, each responsible for a different aspect of SA. The presentation, business logic, and context fabric layers work together to analyse, process, and correlate data.

According to Ahmad et al. [145], an organization's incident response process can be improved by practising situation awareness of the cyberthreat landscape and its broader business context. Park et al. [146,148] provided decision support by analysing threat intelligence to detect mobile device attacks, whereas [146] suggested evaluating threat intelligence for mobile malware from the point of view of SA by using ML algorithms to pull out features that can be used to spot Android malware, whilst the factor analysis of information (FAIR) model was proposed in Ref. [148] to assess the risk associated with IoT devices. They used SA to keep track of what was happening around them in the event of a threat.

#### 4.4.2 Observe, orient, decide, act (OODA) loop model

Boyd developed the OODA in 1996 [149]. Compared to the SA model, the OODA loop was originally designed to help people make decisions. In changing environments like cyberspace, one must make many decisions, so obtaining and maintaining the correct SA is one of the most important requirements. The OODA loop model has four main steps: (i) observe, which means to notice things about the environment; (ii) orient, which means to get a sense of where one is in a certain situation; (iii) decide, which means to decide what the next steps are; and (iv) act, which means to put what has been decided into action.

#### 4.4.3 CSA model

Okolica et al. [150] designed the CSA model in 2009. It suggests a way to build a discovery engine for CSA to find things independently. It consists of three levels: (i) sense, which is the function that includes data collection via sensors; (ii) evaluate the system's ability to synthesize this data into a threat concept that is similar to those already in use; and (iii) assessment by predicting future activities and attacks on the system. For instance, Cheng et al. [99,121,122] presented three studies on APT attack detection using a CSA model. To better understand a cyber situation in IoT systems, an article [99] suggested the APT Alerts and Logs Correlation Method (APTALCM), which provided the edge computing-based framework to deploy APTALCM on IoT systems. While [121] presented APTPMFL, an FL-based APT prediction approach used in 5G-enabled IoT, another article [122] suggested a new way to understand a cyber situation in IoT systems by using recognition of zero-day attack action within APT (CSPAPTM) to learn about zero-day attack activity. According to Alnusair et al. [147], recommendation systems for multimedia data can be based on contextual information and personal preferences.

#### 4.4.4 JDL-DF model

Steinberg et al. [151] designed the JDL-DFM in 1998. It consists of five levels of data processing. Level 0 entails the sub-object data assignment, which collects sensor data. Level 1 combines Level 0 data with sensor data to detect security events. This level identifies, detects, and characterizes computers, adversaries, data flows, and network connections. Level 2 brings together different entities to give an overview of the system or environment. Level 3 predicts future system states or attacks. Level 4 manages sensors and their health. For instance, Ioannou et al. [68] proposed a method for detecting, tracking, and forecasting exfiltration APTs (XAPTs) across the cyber kill chain.

#### 4.4.5 ECSA model

Evancich et al. [152] presented a model that creates SA within computer networks called the ECSA model. It deals with the overall network-level view of the network. As a result, both micro and macro perspectives are used [152]. The macro perspective provides a global network perspective, displaying attacks, network components, and defence choices. The micro perspective emphasizes events or hosts, which serve as the foundation of the macro perspective. ECSA can drill down to a micro level and provide insight into a specific event or host. Thus, analysts can view the status of any specific network element. It can also determine the number of hosts, network elements, and events. The objective is not to visualize the network but to provide analysts and defenders with a tool that enhances their ability to defend it. It differs from CSA because it emphasizes facilitating decision-making, collaboration, and resource management rather than regulating resource access.

ECSA is a proactive approach to cybersecurity that helps organisations assess their current level of security and take steps to improve it. It entails continuously monitoring and analysing data from various sources to identify potential vulnerabilities and take steps to prevent or mitigate potential threats [152]. ECSA consists of four stages: network awareness, threat awareness, operational awareness, and prediction and data fusion. Network awareness includes discovering all relevant components and their potential states. Threat awareness is knowing what kinds of attacks and weaknesses can be used to get into or attack a network. Operational awareness measures the impact of an attack on operational capability. Prediction and data fusion are the prognoses for the future situation. These predictions support decision-making and provide information about possible attacks and countermeasures [152]. Based on a change in network posture, ECSA will provide security analysts with several defence solutions and a corresponding impact analysis. The impact of the posture change on the capabilities of the mission or operation will be highlighted. The information provided to the analyst or defender will assist them in making better decisions or optimizing the defence based on the mission's criteria.

According to this study, the CSA model in APT malware detection is the most widely used model. Most of the time, the Endsley model is the most important way to deal with cybersecurity threats.

## 5 Discussion

From 2012 to 2022, 75 journal articles on APT detection were published; these were reviewed in this study. We drew all the available papers from six digital libraries (ACM, IEEE, ScienceDirect, Scopus, Springer, and Web of Science). We then summarized APT attack detection models based on the abovementioned investigation and concluded that a reliable way to detect APT malware is to watch and analyse network traffic. APT attacks are a hot topic in cybersecurity, and knowing how to spot them is important. Most of the research in this study showed that traditional IDSs did not detect malicious traffic, such as APT attacks, quickly enough. Sections 5.1, 5.2, 5.3 discuss the research challenges, suggestions for future research, and proposed conceptual model.

### 5.1 Research challenges

This section discusses the findings of the research questions. Below is a list of problems that can be considered in the research and technical fields.

The first question concerns ‘the current state of the art concerning detection models for APT attacks’, which researchers frequently used to construct a detection model in primary studies. We classified the detection models into five categories: ML-, deep learning–, static analysis–, statistical analysis–, and other AI-based detection models.

Detection models that rely on ML were the most used, then detection models that rely on deep learning, then detection models based on statistical analysis and detection models based on other AI techniques. Detection models based on static analysis were the least used. However, ML-based detection models have some quality issues, such as computational cost, accuracy, and time. Some studies used deep learning–based detection models to deal with massive amounts of data that required considerable storage and time for decision-making, such as [71,75,82,90,91,98,104,105,108,123,124,126,[128], [129], [130]]. Some studies that used AI-based detection models had problems such as delay, reliability, and computational costs, such as [70,72,74,77,79,122,131,132]. Some studies that used static analysis–based detection models had problems such as data flow and taint analysis [81,94,101]. Other studies used statistical analysis–based detection models, which have problems in areas such as time and accuracy; for examples of these studies, see Refs. [65,68,69,73,85,87,99,111,116,127].

The second question relates to ML techniques often used to build models for object detection. Clustering and ensemble learning has been used in many models to identify APT attacks. However, clustering is ineffective because dataset size can make the method problematic because of the time complexity that data set size can introduce; thus, the results may not be accurate, as was the case in Refs. [67,86,93,106,107,118]. Some studies used ensemble learning, which combines more than one algorithm to achieve the best results, as was done in Refs. [83,89,97,100,102,112,120,125]. RF is not good for making predictions in real-time. In general, this algorithm is quick to train but takes a long time to make predictions, which can be seen in Refs. [80,96,117,119].

A decision tree is ineffective because the detection results are inaccurate, as seen in Refs. [66,113,114]. FL is a model for large-scale distributed learning that deals with two major problems: how to efficiently use data from many different users and how to protect the privacy of users who are taking part, as is made clear in Refs. [76,78,121]. Naïve Bayes might not be as effective in handling more complex classification problems. It can work only when the features are independent, such as [95,110]. PHC can adapt to changing attack patterns and detect previously unknown attacks. However, it can be computationally expensive and may require a lot of training data. It may also be ineffective against attacks designed to evade detection, such as those in Refs. [70,132].

The AVF algorithm gives less precision and a high recall value [84]. The GAF algorithm is used to detect APT on mobile devices [88]. LR needs a linear relationship between the input and output variables, so it does not work well with complex data sets, such as those in Ref. [92]. HMM requires a long time to be trained and thus can be quite slow, like in the results of [103]. SVM is unsuitable for big data sets because it takes a long time to train, like in Ref. [109]. ELM is much faster in training but cannot encode more than one abstraction layer, so it can be very slow to provide an evaluation, as seen in Ref. [115].

The third question is related to SA models in cybersecurity. In this question, only 10 studies discussed SA models. Three SA models used by researchers are the SA model, the CSA model, and the Joint Directors of Laboratories data fusion (JDL-DF) model. Four studies introduced APT attack detection: one on the computer [68] using JDL-DF and the rest on IoT [99,121,122] using CSA. Whereas the remaining six studies discussed the detection of traditional attacks, several other studies [[143], [144], [145], [146],^148^] used Endsley's SA and [147] used CSA.

### 5.2 Recommendations

In this study, we identified some of the problems with detecting APT attacks and gave an overview of the work that can be done to solve these problems. Due to the complex nature of APT attacks, they can be uncovered only by uncovering the many stages of the APT life cycle. Some previous approaches have attempted to detect potential APT attacks, and most of the studies focused on detecting one stage of the APT, such as C&C [88,120]. Thus, the current APT detection systems need to improve in several aspects. Detecting an APT technique is unlike detecting an APT attack. Thus, we recommend building systems that detect all the APT stages identifiable through network traffic, such as delivery, C&C, and exfiltration.

Another issue is that traditional IDS cannot find new patterns of APT of malicious traffic with unknown, unusual, or abnormal behaviour, such as zero-day attacks, with a high detection rate and a low rate of false positives [89]. One of the most promising ways to do this while simultaneously minimizing false negatives and false positives is to use ML. Thus, we recommend applying ensemble learning techniques to improve the APT attack detection model. Ensemble learning combines multiple approaches to overcome their limitations and produce a more accurate model. It leverages the strengths of each approach to make better overall decisions. This makes it more effective than using a single ML technique. Another issue is the IDSs alone are not sufficient to detect and predict multiphase, long-duration attacks early in their life cycle, such as APT attacks [67,90]. Therefore, IDSs require support through continuous monitoring and assisting decision-makers in making the best decision. Thus, we recommend using the ECSA model to support IDS, which helps to optimize the detection and prediction of APT attacks in the early stages by correlating the alerts over a long time.

As a result, we recommend a continuous monitoring conceptual model, the Effective Cyber Situational Awareness Model to Detect and Predict Mobile APTs (ECSA-tDP-MAPT) to effectively detect and predict mobile APTs based on network traffic, which includes using ML techniques to train and test the model. In addition, the ECSA-tDP-MAPT model provides constant monitoring and analysis of data from different sources to find possible weaknesses and take steps to stop or lessen the impact of a possible mobile APT.

### 5.3 Proposed ECSA-tDP-MAPT conceptual model

A wireless local area network (WLAN) is a network of wireless devices that connects two or more devices using wireless communication to form a local area network (LAN) within a limited area, such as a company, school, computer lab, campus, or bank. A WLAN consists of user devices and users of WLANs operate many devices, such as mobile devices, laptops, and PDAs, in addition to routers, access points, switches, and server devices.

In this section, we propose a conceptual model for use in WLAN architecture, especially on mobile devices, to detect mobile APT, the ECSA-tDP-MAPT model. This conceptual model is innovative and promising for combating APT. It enables security experts to make accurate decisions regarding APT-related suspicious incident detection. It is a multistage and comprehensive concept for APT detection and prediction based on continuous network traffic monitoring. Also, this model follows the NIST cybersecurity framework. Cyber-cognitive situation awareness (CCSA) is implemented within the NIST framework. Cognitive security is derived from cognitive science and focuses heavily on adopting AI technologies patterned on human thought processes to detect threats and protect physical and digital systems [153]. AI techniques such as ML will be used to classify APT-based TTP or normal and help people make accurate decisions.

CCSA is defined as self-awareness capable of acquiring the following properties during execution: self-adaptive, auto-predictive, and auto-reflective. ‘Self-adaptive’ means capable of proactively adapting to its environment to continue achieving its operational goals, ‘auto-predictive’ means it can predict how the dynamic change caused by possible adaptation actions will affect the system, and ‘auto-reflective’ means it knows its software architecture, hardware infrastructure, and execution environment so that it can meet its operational goals. ECSA is a CCSA model. It provides a holistic view of situation awareness within a network by providing better intelligence around the network status and helping to capture various types of threats, analyse them, and work to detect and reduce them [152]. The conceptual model aims to detect and predict mobile APT more accurately and make people aware of their environment.

The conceptual model further applies offensive and defensive security measures, which are key cybersecurity concepts. The offensive track deploys a proactive approach to security through ethical hacking. Offensive cybersecurity teams actively test the network's defences and provide valuable insights into an organization's cybersecurity posture [154]. On the other hand, the defensive track uses a reactive approach to security that focuses on prevention, detection, and response to attacks. Defensive measures include firewalls, intrusion prevention systems, IDS, virtual private networks, and strong passwords [154]. A general track also uses a mix of offensive and defensive tactics to provide cybersecurity.

**Fig. 13:**
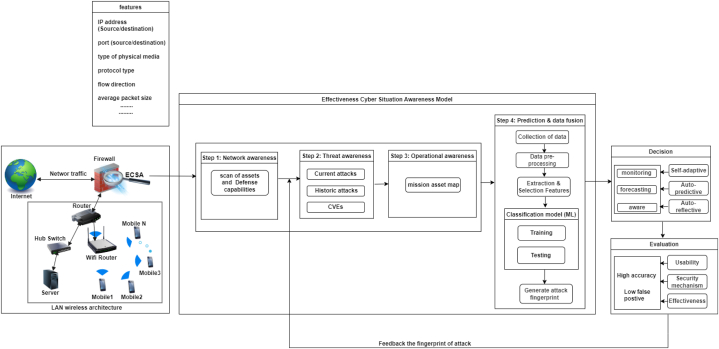
ECSA-tDP-MAPT conceptual model.

As shown in Fig. 13, the ECSA model has four stages: network awareness, threat awareness, operational awareness, and prediction and data fusion. These stages are explained in the sections that follow.

#### 5.3.1 Network awareness

Network awareness means understanding the current state and condition of all the elements that make up the network – servers, hardware, cabling, and power. A network requires a suitable configuration. Often, assets are interdependent, and some may have redundancies. Network awareness includes recovery time that can result from a reboot, hardware failure, or patching. Achieving SA requires the discovery or enumeration of assets and defence capabilities.

The first step in achieving network awareness is the discovery or enumeration of assets. It involves identifying and keeping track of the various components in a large and complex network. However, this can be challenging because the network constantly changes, which requires refreshing the network scan. Technologies used for discovery may include network scanners such as Nmap, Zmap, runZero, and Nessus. A network scanner is designed for host discovery, port scanning, version detection, and operating system detection; also, it can provide additional details about the targeted device, such as the device model and MAC-layer address.

Defence capabilities refer to the options available to defenders to protect their network against attacks. It is the second step to gaining network awareness. Defence capabilities have inference and anti-inference tools. While an attack is happening, defenders can use these tools to make inferences about the capabilities and intentions of the attackers. This information can then assess the impact of defence postures and predict how the attacker will react to network defence changes. For instance, shadow honeypots can be situated at attack pressure points to determine how an attacker responds to them. It is an unbalanced exchange because the attacker must expend resources to comprehend the shadow honeypot but receives no valuable information in return. On the other hand, the defenders will observe the complexity of the attacks with minimal effort.

#### 5.3.2 Threat awareness

Threat awareness involves understanding the current and historical attacks a network may be vulnerable to and any flaws or holes in the current network based on several exploit sources, such as common vulnerabilities and exposures CVEs. Threat awareness aims to identify attack vectors that pose a risk to network assets. To acquire information about the threats and attacks that face network traffic, one should combine knowledge about the attacker's position, capability, and posture; indicators and warnings; threat identification and detection; and vulnerabilities and how attacks may occur in the network setting. Graphical models can effectively represent probable attack paths in an enterprise network and enable static and dynamic analysis of the network's security posture.

Attack graphs (AGs) are used to analyse the relations between attack events and evaluate the probable impact of multistep attacks on a network. However, current AG techniques have limitations in evaluating the impact on high-level missions and need to be more scalable for large-scale networks. To address the constraints of current AG techniques, a team has developed an efficient AG model with three types: type AG (TAG), network attack graph (NAG), and real-time attack graph (RAG). These models have been incorporated into a software toolkit called NIRVANA, which can automatically generate TAGs and NAGs for static security analysis and RAGs for dynamic security analysis and damage assessment. For example, if an IDS alert indicates a possible network attack, a RAG can be generated to analyse the attack in real-time and assess the damage. This information can then be used to improve the network's posture and prevent future attacks.

#### 5.3.3 Operational awareness

Threats or attacks can affect tasks or processes in networks. Engaging defences may affect services and their availability. For instance, the network offers various services (email, authentication, etc.). Changing the network's posture or deploying defences could affect these services. Operational or mission awareness involves breaking down complex missions into manageable tasks. A mission asset map identifies necessary network components and cyber assets, improving cybersecurity operations. Tasks can be primitive or compound and are interrelated by dependencies and constraints. Network components are prioritized based on their criticality for mission assurance using an algorithm such as the analytic hierarchy process (AHP) for risk analysis and cyber asset prioritization.

#### 5.3.4 Prediction and data fusion

The last stage includes the following steps:

(i) Data collection – model collects the data by putting together all the information from the first three steps: network awareness, threat awareness, and operational awareness.
(ii) Data preprocessing – involves removing duplicates and missing values and normalizing the data because the collected data has a wide range of numeric values. Normalization reduces the range of the values to a common scale. In addition, normalization speeds up the model training stage.
  (iii) Extraction and selection of features – extracts features to detect mobile APT based on network traffic, such as IP address (source/destination), port (source/destination), protocol type, flow direction, average packet size, the total number of visits, addresses with the same domain, and resource record time to live. Features related to the APT attack are extracted and selected with the help of one of the tools, such as the CICFlowMeter tool.
(iv) Classification model using ML techniques – we constructed a classifier for nonsuspicious or suspicious behaviour using multiple classification techniques based on the premise that APT-infected traffic tends to exhibit anomalous characteristics. This technique will train and test this suspicious behaviour to detect unknown APT attacks. It will generate a new APT fingerprint (APT-based TTP) if detected. The fingerprint is intended to determine how a system can be attacked and identify its weak points. As a decision manager, the maximum-security measure is taken for the risk path through threat evaluation feedback, thus reducing the probability of being attacked by APT. Thus, the model can develop situation-based awareness based on self-adaptive, auto-predictive, and auto-reflective traits.

## 6 Conclusions and future work

This study summarizes the most recent techniques and offers an in-depth overview of the methods used to identify APT malware. This article examined 75 articles published from 2012 to 2022 and examines the types of detection techniques, how empirical experiments are done, how APT malware can be found using different detection techniques, and how well different models can find APT malware. Based on the studies reviewed, we derived the following conclusions and implications about using ML to find APT malware:

(i) The ML technique is the most commonly used detection mechanism to detect APT malware.
(ii) Clustering, extreme learning, RF, decision tree, FL, naïve Bayes, SVM, LR, genetic function approximation, AVF, HMM, and ELM were the most used ML techniques in the studies, and extreme learning was the most widely used technique for APT attack detection.
(iii) The results related to detection mechanisms show that the traditional IDSs are ineffective at detecting APT malware in real-time. This is because the behaviour of APT continuously changes.

(iv) A few articles used SA models to detect APT malware by monitoring and analysing the network traffic using a CSA model. We conclude that traditional IDSs still face some challenges in detecting APT malware through the monitoring and analysis of network traffic. To mitigate the challenge IDSs faces, we suggest some rules that will help find new ways for detection systems to detect APT malware and ease the problem. For example, an SA model that uses two or more ML techniques could be helpful.

We have also proposed a conceptual model, ECSA-tDP-MAPT, to detect and predict mobile APTs based on network traffic effectively. In the future, we will follow the insights gained from this SLR to facilitate identifying APT malware by monitoring and analysing malicious traffic that is encrypted and unencrypted.

## Declarations

### Author contribution statement

All authors listed have significantly contributed to the development and the writing of this article.

### Funding statement

This work is supported by the Ministry of Higher Education Malaysia under the Fundamental Research Grant Scheme with project code FRGS/1/2020/ICT07/USM/02/2. Also, the first author would like to thank Mustansiriyah University for its scholarship support for studying in Malaysia.

### Data availability statement

No data was used for the research described in the article.

## Declaration of competing interest

The authors declare that they have no known competing financial interests or personal relationships that could have appeared to influence the work reported in this paper.

## References

[1] O'Brien R.T., Ditfurth J.V., Mrics H.A. (2018). https://www2.deloitte.com/global/en/pages/real-estate/articles/future-real-estate-data-new-gold.html.

[2] (2022). FinancesOnline.

[3] Stallings W., Slyke R.V. (2001).

[4] Alwahedi S., Ali M.A., Oloko F.I., Woon W.L., Aung Z. (2016). Proc. LNICST, MONAMI 2016.

[5] Wilmer H.H., Sherman L.E., Chein J.M. (2017). Smartphones and cognition: a review of research exploring the links between mobile technology habits and cognitive functioning. Front. Psychol..

[6] Alshamrani A., Myneni S., Chowdhary A., Huang D. (2019). A survey on advanced persistent threats: techniques, solutions, challenges, and research opportunities. IEEE Commun. Surv. Tutor..

[7] Lelli A. (2013). https://www.symantec.com/connect/blogs/remote-access-tool-takes-aim-android-apk-binder.

[8] Unuchek R. (2017). https://securelist.com/dvmap-the-first-android-malware-with-code.

[9] (2022). Statista.

[10] (2022). Statista.

[11] Chuan B.L.J., Singh M.M., Shariff A.R.M. (2018). Proc.

[12] (2022). MITRE ATT&CK.

[13] Lashkari A.H., Gil G.D., Mamun M.S.I., Ghorbani A.A. (2017). Proc. 3rd ICISSP.

[14] Li Y., Huang G.Q., Wang C.Z., Li Y.C. (2019). Analysis framework of network security situational awareness and comparison of implementation methods. EURASIP J. Wirel. Commun. Netw..

[15] Hussain S., B Ahmad M., Ghouri S.S.U. (2021). Advance persistent threat–A systematic review of literature and meta-analysis of threat vectors. Adv. Intell. Syst. Comput..

[16] Jabar T., Singh M.M. (2022). Exploration of mobile device behavior for mitigating advanced persistent threats (APT): a systematic literature review and conceptual framework. Sensors.

[17] Talib M.A., Nasir Q., Nassif A.B., Mokhamed T., Ahmed N., Mahfood B. (2022). APT beaconing detection: a systematic review. Comput. Secur..

[18] Kotenko I., Gaifulina D., Zelichenok I. (2022). Systematic literature review of security event correlation methods. IEEE Access.

[19] Khalid M.N.A., Al-Kadhimi A.A., Singh M.M. (2023). Recent developments in game-theory approaches for the detection and defense against advanced persistent threats (APTs): a systematic review. Mathematics.

[20] Jeun I., Lee Y., Won D.A. (2012). Proc.CCIS.

[21] FireEye (2018). https://library.cyentia.com/report/report_001522.html.

[22] Coopers Pricewaterhouse, Hopper Operation Cloud (2017). https://www.pwc.co.uk/cyber-security/pdf/pwc-uk-operation-cloud-hopper-report-april-2017.pdf.

[23] FireEye A.P.T.4 (2019). https://content.fireeye.com/apt-41/rpt-apt41/.

[24] Zulkefli Z., Singh M.M., Mohd Shariff A.R., Samsudin A. (2017). Typosquat cyber crime attack detection via smartphone. Procedia Comput. Sci..

[25] (2023). Statista.

[26] Langner R. (2011). Stuxnet: dissecting a cyberwarfare weapon. IEEE Secur. Priv..

[27] Cluley G. (2014). https://www.ptsecurity.com/ww-en/analytics/pt-esc-threat-intelligence/apt-cloud-atlas-unbroken-threat/.

[28] (2018). Lookout, Stealth Mango & Tangelo, Microsoft, Headquartered in San Francisco, USA.

[29] Johnson A.L. (2016).

[30] Ferrer Z., Ferrer M.C. (2010).

[31] Proofpoint (2017). https://www.proofpoint.com/us/threat-insight/post/credential-phishing-and-android-banking-trojan-combine-austrian-mobile-attacks.

[32] Firsh A., Baumgartner K., Bartholomew B., Twosail J.U.N.K. (2020). https://securelist.com/ios-exploit-chain-deploys-lightspy-malware/96407.

[33] Dedola G. (2020). https://securelist.com/transparent-tribe-part-1/98127.

[34] Global Research & Analysis Team (2020). https://securelist.com/mata-multi-platform-targeted-malware-framework/97746.

[35] Kwiatkowski I., Delcher P., Yamout M. (2020). https://securelist.com/deathstalker-mercenary-triumvirate/98177.

[36] Firsh A. (2018). https://securelist.com/whos-who-in-the-zoo/85394.

[37] Lab Kaspersky, Global Research, Analysis Team (2019). https://securelist.com/new-finspy-ios-and-android-implants-revealed-itw/91685.

[38] Gostev A., Kamluk V. (2016). https://securelist.com/adwind-faq/73660.

[39] Lab Kaspersky, Global Research, Analysis Team (2014). https://securelist.com/hackingteam-2-0-the-story-goes-mobile/63693.

[40] Saad G., Hasbini M.A. (2015). https://securelist.com/the-desert-falcons-targeted-attacks/68817.

[41] Zulkefli Z., Singh M.M. (2020). Sentient-based access control model: a mitigation technique for advanced persistent threats in smartphones. J. Inf. Secur. Appl..

[42] Tatam M., Shanmugam B., Azam S., Kannoorpatti K. (2021). A review of threat modelling approaches for APT-style attacks. Heliyon.

[43] Alcaraz C. (2018). Cloud-assisted dynamic resilience for cyber-physical control systems. IEEE Wireless Commun..

[44] Martin Locked (2015). Gaining the Advantage: Applying Cyber Kill Chain® Methodology to Network Defense.

[45] Souppaya M., Scarfone K. (2016).

[46] Schneier B. (1999). https://www.schneier.com/academic/archives/1999/12/attack_trees.html.

[47] Al-Shaer R., Spring J.M., Christou E. (2020). IEEE Conf. Commun.Netw. Secur..

[48] Meucci M., Andrew M. (2014).

[49] Gore R., Padilla J., Diallo S. (2017). Markov chain modeling of cyber threats. J. Def. Mod. Simul. Appl. Method. Techn..

[50] MITRE, CAPEC VIEW (2019). Mechanisms of Attack.

[51] Wynn J.E. (2013). https://www.mitre.org/publications/technical-papers/presentation-threat-assessment-remediation-analysis-tara-methodology.

[52] Carreon C. (2018). https://www.recordedfuture.com/diamond-model-intrusion-analysis.

[53] Tankard C. (2011). Advanced persistent threats and how to monitor and deter them. Network Security.

[54] Giura P., Wang W. (2012). Proc. International Conference on Cyber Security.

[55] Hong J., Liu C., Govindarasu M. (2014). Integrated anomaly detection for cyber security of the Substations. IEEE Trans. Smart Grid.

[56] Siddiqui S., Khan M.S., Ferens K., Kinsner W. (2016). Proc. ACM on International Workshop on Security and Privacy Analytics.

[57] Marchetti M., Pierazzi F., Guido A., Colajanni M. (2016). Proc. 8th International Conference on Cyber Conflict (CyCon).

[58] Rubio J.E., Alcaraz C., Lopez J. (2017).

[59] Rubio J.E., Roman R., Alcaraz C., Zhang Y. (2018).

[60] Elrawy M.F., Awad A.I., Hamed H.F.A. (2018). Intrusion detection systems for IoT-based smart environments: a survey. J. Cloud Comput..

[61] Khraisat A., Gondal I., Vamplew P., Kamruzzaman J. (2019). Survey of intrusion detection systems: techniques, datasets and challenges. Cybersecurity.

[62] Mukherjee B., Heberlein L.T., Levitt K.N. (1994). Network intrusion detection. IEEE Netw.

[63] Yao J., Fan X., Cao N. (2020). Proc. International Symposium on Cyberspace Safety and Security.

[64] Budgen D., Brereton P. (2006). Proc. 28th Int. Conf. Softw.

[65] Yan X., Zhang J.Y. (2013). Early detection of cyber security threats using structured behavior modeling. ACM Trans. Inf. Syst. Secur..

[66] Zhao G., Xu K., Xu L., Wu B. (2015). Detecting APT malware Infections based on malicious DNS and traffic analysis. IEEE Access.

[67] Cho D.X., Nam H.H. (2019). A method of monitoring and detecting APT attacks based on unknown domains. Prog. Commun. Sci..

[68] Ioannou G., Louvieris P., Clewley N. (2019). A Markov multi-phase transferable belief model for cyber situational awareness. IEEE Access.

[69] Khosravi M., Ladani B.T. (2020). Alerts correlation and causal analysis for APT based cyber attack detection. IEEE Access.

[70] Xiao L., Xu D., Mandayam N.B., Poor H.V. (2018). Attacker-centric view of a detection game against advanced persistent threats. IEEE Trans. Mobile Comput..

[71] Rahman Z., Yi X., Khalil I. (2022). Blockchain based AI-enabled industry 4.0 CPS protection against advanced persistent threat. IEEE Internet Things J..

[72] Ghafir I., Prenosil V., Hammoudeh M., Baker T., Jabbar S., Khalid S., Jaf S. (2018). BotDet: a system for real time botnet command and control traffic detection. IEEE Access.

[73] Xiong C., Zhu T., Dong W., Ruan L., Yang R., Cheng Y., Chen Y., Cheng S., Chen X. (2020). CONAN: a practical real-time APT detection system with high accuracy and efficiency. IEEE Trans. Dependable Secure Comput..

[74] Ma Z., Li Q., Meng X. (2019). Discovering suspicious APT families through a large-scale domain graph in information-centric IoT. IEEE Access.

[75] Joloudari J.H., Haderbadi M., Mashmool A., GhasemiGol M., Band S.S., Mosavi A. (2020). Early detection of the advanced persistent threat attack using performance analysis of deep learning. IEEE Access.

[76] Xu S., Qian Y., Hu R.Q. (2020). Edge intelligence assisted gateway defense in cyber security. IEEE Netw.

[77] Li H., Wu J., Xu H., Li G., Guizani M. (2021). Explainable intelligence-driven defense mechanism against advanced persistent threats: a Joint edge game and AI approach. IEEE Trans. Dependable Secure Comput..

[78] Taheri R., Shojafar M., Alazab M., Tafazolli R., Fed-IioT (2020). A robust federated malware detection architecture in Industrial IoT. IEEE Trans. Ind. Inf..

[79] Khan H.A., Sehatbakhsh N., Nguyen L.N., Callan R.L., Yeredor A., Prvulovic M., Zajic A. (2019). IDEA: intrusion detection through electromagnetic-signal analysis for critical embedded and cyber- physical systems. IEEE Trans. Dependable Secure Comput..

[80] Niu W., Xiao j., Zhang X., Zhang x., Du X., Huang X., Guizani M. (2020). Malware on internet of UAVs detection combining string matching and Fourier transformation. IEEE Internet Things J..

[81] Dube T.E., Raines R.A., Grimaila M.R., Bauer K.W., Rogers S.K. (2012). Malware target recognition of unknown threats. IEEE Syst. J..

[82] Min B., Yoo J., Kim S., Shin D., Shin D. (2021). Network anomaly detection using memory-augmented deep autoencoder. IEEE Access.

[83] Moustafa N., Choo K.K.R., Radwan I., Camtepe S. (2019). Outlier Dirichlet mixture mechanism: adversarial statistical learning for anomaly detection in the fog. IEEE Trans. Inf. Forensics Secur..

[84] Berrada G., Cheney J., Benabderrahmane S., Maxwell W., Mookherjee H., Theriault A., Wright R. (2020). A baseline for unsupervised advanced persistent threat detection in system-level provenance. Future Generat. Comput. Syst..

[85] Lajevardi A.M., Amini M. (2019). A semantic-based correlation approach for detecting hybrid and low-level APTs. Future Gener. Comput. Syst..

[86] Marchetti M., Pierazzi F., Colajanni M., Guido A. (2016). Analysis of high volumes of network traffic for advanced persistent threat detection. Comput. Network..

[87] Friedberg I., Skopik F., Settanni G., Fiedler R. (2015). Combating advanced persistent threats: from network event correlation to Incident detection. Comput. Secur..

[88] Xiang Z., Guo D., Li Q. (2020). Detecting mobile advanced persistent threats based on large-scale DNS logs. Comput. Secur..

[89] Ghafir I., Hammoudeh M., Prenosil V., Han L., Hegarty R., Rabie K., Aparicio-Navarro F.J. (2018). Detection of advanced persistent threat using machine- learning correlation analysis. Future Generat. Comput. Syst..

[90] Shang L., Guo D., Ji Y., Li Q. (2021). Discovering unknown advanced persistent threat using shared features mined by neural networks. Comput. Network..

[91] Fang Y., Wang C., Fang Z., Huang C. (2022). LMTracker: lateral movement path detection based on heterogeneous graph embedding. Neurocomputing.

[92] Burnap P., French R., Turner F., Jones K. (2018). Malware classification using self organising feature maps and machine activity data. Comput. Secur..

[93] Zimba A., Chen H., Wang Z., Chishimba M. (2020). Modeling and detection of the multi-stages of advanced persistent threats attacks based on semi- supervised learning and complex networks characteristics. Future Generat. Comput. Syst..

[94] Santos I., Brezo F., Ugarte-Pedrero X., Bringas P.G. (2013). Opcode sequences as representation of executables for data-mining-based unknown malware detection. Inf. Sci..

[95] Y. Ahmed, A.T. Asyhari, M.A. Rahman, A cyber kill chain approach for detecting advanced persistent Threat, Comput. Mater. Continua (CMC) 67 (2021), 2497-2513, 10.32604/cmc.2021.014223.

[96] Xuan C.D., Duong D., Dau H.X. (2021). A multi-layer approach for advanced persistent threat detection using machine learning based on network traffic. J. Intell. Fuzzy Syst..

[97] Javed S.H., Ahmad M.B., Asif M., Almotiri S.H., Masood K., Al Ghamdi M.A. (2022). An intelligent system to detect advanced persistent threats in industrial internet of things (I-IoT). Electron. Times.

[98] Xuan C.D., Nguyen H.D., Dao M.H. (2020). APT attack detection based on flow network analysis techniques using deep learning. J. Intell. Fuzzy Syst..

[99] Cheng X., Zhang J., Chen B. (2019). Cyber situation comprehension for IoT systems based on apt alerts and logs Correlation. Sensors.

[100] Chu W.L., Lin C.J., Chang K.N. (2019). Detection and classification of advanced persistent threats and attacks using the support vector machine. Appl. Sci..

[101] Chakkaravarthy S.S., Vaidehi V., Rajesh P. (2018). Hybrid analysis technique to detect advanced persistent threats. Int. J. Intell. Inf. Technol..

[102] Alqahtani M., Mathkour H., Ismail M.M.B. (2020). IoT botnet attack detection based on optimized extreme gradient boosting and feature selection. Sensors.

[103] Brogi G. (2018).

[104] Hwang C., Kim D., Lee T. (2020). Semi-supervised based unknown attack detection in EDR environment. KSII Trans. Inter. Inf. Syst..

[105] Xuan C.D., Dao H.M. (2021). A novel approach for APT attack detection based on combined deep learning Model. Neural Comput. Appl..

[106] Vatamanu C., Gavriluţ D., Benchea R. (2012). A practical approach on clustering malicious PDF documents. J. Comput. Virol..

[107] Lu J., Chen K., Zhuo Z., Zhang X. (2019). A temporal correlation and traffic analysis approach for APT attacks detection. Cluster Comput..

[108] Demertzis K., Iliadis L., Tziritas N., Kikiras P. (2020). Anomaly detection via block chained deep learning smart contracts in industry 4.0, Neural Comput. Appl.

[109] Wang X., Liu Q., Pan Z., Pang G. (2020). APT attack detection algorithm based on spatio-temporal association analysis in industrial network. J. Ambient Intell. Hum. Comput..

[110] Panahnejad M., Mirabi M. (2022). APT-Dt-KC: advanced persistent threat detection based on kill-chain model. J. Supercomput..

[111] Lajevardi A.M., Amini M. (2021). Big knowledge-based semantic correlation for detecting slow and low-level advanced persistent threats. J. Big Data.

[112] Sharma P.K., Moon S.Y., Moon D., Park J.H., DFA-AD (2017). A distributed framework architecture for the detection of advanced persistent threats. J.Clus. Comp..

[113] Moon D., Im H., Kim I., Park J.H., DTB-IDS (2017). An intrusion detection system based on decision tree using behavior analysis for preventing APT attacks. J. Supercomput..

[114] Moon D., Pan S.B., Kim I. (2016). Host-based intrusion detection system for secure human-centric computing. J. Supercomput..

[115] Shi Y., Chen G., Li J. (2018). Malicious domain name detection based on extreme machine learning. Neur. Process. Letters.

[116] Navarro J., Legrand V., Deruyver A., Parrend P. (2018). OMMA: open architecture for operator-guided monitoring of multi-step attacks. EURASIP J. Inf. Secur..

[117] Bolton A.D., Anderson-Cook C.M. (2017). APT malware static trace analysis through bigrams and graph edit Distance. Stat. Anal. Data Min..

[118] Zhang R., Huo Y., Liu J., Weng F. (2017). Constructing APT attack scenarios based on intrusion kill chain and fuzzy clustering. Secur. Commun. Netw..

[119] Xuan C.D., Duong L.V., Nikolaevich T.V. (2020). Detecting C&C server in the APT attack based on network traffic using machine learning. Int. J. Adv. Comput. Sci. Appl..

[120] Niu W., Zhang X., Yang G., Zhu J., Ren Z. (2017). Identifying APT malware domain based on mobile DNS Logging. Math. Probl Eng..

[121] Cheng X., Luo Q., Pan Y., Li Z., Zhang J., Chen B. (2021). Predicting the APT for cyber situation comprehension in 5G- enabled IoT scenarios based on differentially private federated learning. Secur. Commun. Network..

[122] Cheng X., Zhang J., Tu Y., Chen B. (2022). Cyber situation perception for Internet of Things systems based on zero‐day attack activities recognition within advanced persistent threat. Concurrency Comput. Pract. Ex..

[123] Bodström T., Hämäläinen T. (2019). A novel deep learning stack for APT detection. Appl. Sci..

[124] Xuan C.D., Huong D.T. (2022). A new approach for APT malware detection based on deep graph network for endpoint systems. Appl. Intell..

[125] Al-Saraireh J., Masarweh A. (2022). A novel approach for detecting advanced persistent threats. Egyp. Inform. J..

[126] Niu W., Zhou J., Zhao Y., Zhang X., Peng Y., Huang C. (2022). Uncovering APT malware traffic using deep learning combined with time sequence and association analysis. Comput. Secur..

[127] Yang J., Zhang Q., Jiang X., Chen S., Yang F. (2022). POIROT: causal correlation aided semantic analysis for advanced persistent threat detection. IEEE Trans. Dependable Secure Comput..

[128] Xuan C.D., Huong D.T., Duong D. (2022). New approach for APT malware detection on the workstation based on process profile. J. Intell. Fuzzy Syst..

[129] Xuan C.D., Huong D.T., Nguyen T. (2022). A novel intelligent cognitive computing-based APT malware detection for Endpoint systems. J. Intell. Fuzzy Syst..

[130] Xuan C.D., Duong D. (2022). Optimization of APT attack detection based on a model combining ATTENTION and deep learning. J. Intell. Fuzzy Syst..

[131] Rubio J.E., Roman R., Alcaraz C., Zhang Y. (2019). Tracking APTs in industrial ecosystems: a proof of concept. J. Comput. Secur..

[132] Min M., Xiao L., Xie C., Hajimirsadeghi M., Mandayam N.B. (2018). Defense against advanced persistent threats in dynamic cloud storage: a colonel blotto game approach. IEEE Internet Things J..

[133] Abdullayeva F.J. (2021). Advanced persistent threat attack detection method in cloud computing based on autoencoder and softmax regression algorithm. Array.

[134] Sarker I.H. (2021). Machine learning: algorithms, real-world applications and research directions. SN Comp. Sci..

[135] Jaderberg M., Mnih V., Czarnecki W.M., Schaul T., Leibo J.Z., Silver D., Kavukcuoglu K. (2016). Reinforcement learning with unsupervised auxiliary tasks. arXiv preprint arXiv:1611.05397.

[136] Dai D., Boroomand S. (2021). A review of artificial intelligence to enhance the security of big data systems: state-of-art, methodologies, applications, and challenges. Arch. Comput. Methods Eng..

[137] (2022). Reciprocity.

[138] Chen W., Helu X., Jin C., Zhang M., Lu H., Sun Y., Tian Z. (2020). Advanced persistent threat organization identification based on software gene of malware. Trans. Emerg. Telecommun. Techn..

[139] STOUT (2022). https://www.stout.com/en/insights/article/can-machine-learning-help-cybersecurity-systems.

[140] Dong X., Yu Z., Cao W., Shi Y., Ma Q. (2020). A survey on ensemble learning. Front. Comput. Sci..

[141] Pahi T., Leitner M., Skopik F. (2017). Proc. 3rd International¸ Conference on Information Systems Security and Privacy (ICISSP’17).

[142] Alavizadeh H., Jaccard J.J., Yusuf Enoch S., Al-Sahaf H., Welch I., Camtepe S.A., Ki D.S., A survey on threat situation awareness systems: framework, techniques, and insights, *arXiv preprint arXiv:2110.15747* (2021), doi:10.48550/arXiv.2110.15747.

[143] Xu G., Cao Y., Ren Y., Li X., Feng Z. (2017). Network Security Situation Awareness based on Semantic Ontology and User-Defined Rules for Internet of Things. IEEE Access.

[144] Rapuzzi R., Repetto M. (2018). Building situational awareness for network beyond the security perimeter model. Future Generat. Comput. Syst..

[145] Ahmad A., Maynard S.B., Desouza K.C., Kotsias J., Whitty M.T., Baskerville R.L. (2021). How can organizations develop situation awareness for incident response: a case study of management practice. Comput. Secur..

[146] Park M., Seo J., Han J., Oh H., Lee K. (2018). Situational awareness framework for threat intelligence measurement of android malware. J. Wirel. Mob. Netw. Ubiquitous Comput. Dependable Appl. (JoWUA).

[147] Alnusair A., Zhong C., Rawashdeh M., Hossain M.S., Alamri A. (2017). Context-aware multimodal recommendations of multimedia data in cyber situational awareness. Multimed. Tool. Appl..

[148] Park M., Han J., Oh H., Lee K. (2019). Threat assessment for android environment with connectivity to IoT devices from the perspective of situational awareness, Wirel. Commun. Mob. Comp..

[149] Boyd J.R. (1996). The essence of winning and losing. Unpub. Lect. Notes.

[150] Okolica J., Mcdonald J.T., Peterson G.L., Mills R.F., Haas M.W. (2009). Proc. 2nd Cyberspace Res.Workshop, Shreveport, LO, USA.

[151] Steinberg A.N., Bowman C.L., White F.E. (1998). Proc. NATO/IRIS Conference.

[152] Evancich N., Lu Z., Li J., Cheng Y., Tuttle J., Xie P. (2014).

[153] Andrade R.O., Yoo S.G. (2019). Cognitive security: a comprehensive study of cognitive science in cybersecurity. J. Inf. Secur. Appl..

[154] Ajmal A.B., Shah M.A., Maple C., Asghar M.N., Islam S.U. (2021). Offensive security: towards proactive threat hunting via adversary emulation. IEEE Access.

